# City-level impact of extreme temperatures and mortality in Latin America

**DOI:** 10.1038/s41591-022-01872-6

**Published:** 2022-06-27

**Authors:** Josiah L. Kephart, Brisa N. Sánchez, Jeffrey Moore, Leah H. Schinasi, Maryia Bakhtsiyarava, Yang Ju, Nelson Gouveia, Waleska T. Caiaffa, Iryna Dronova, Saravanan Arunachalam, Ana V. Diez Roux, Daniel A. Rodríguez

**Affiliations:** 1 Urban Health Collaborative, Drexel Dornsife School of Public Health, Philadelphia, PA, USA; 2 Department of Epidemiology and Biostatistics, Drexel Dornsife School of Public Health, Philadelphia, PA, USA; 3 Department of Environmental and Occupational Health, Drexel Dornsife School of Public Health, Philadelphia, PA, USA; 4 Department of City and Regional Planning and Institute for Transportation Studies, University of California, Berkeley, CA, USA; 5 School of Architecture and Urban Planning, Nanjing University, Nanjing, China; 6 Department of Preventive Medicine, University of Sao Paulo Medical School, Sao Paulo, Brazil; 7 Observatório de Saúde Urbana de Belo Horizonte, Universidade Federal de Minas Gerais, Belo Horizonte, Brazil; 8 Department of Environmental Science, Policy & Management, & Department of Landscape Architecture & Environmental Planning, University of California, Berkeley, CA, USA; 9 Institute for the Environment, University of North Carolina at Chapel Hill, Chapel Hill, NC, USA

## Abstract

Climate change and urbanization are rapidly increasing human exposure to extreme ambient temperatures, yet few studies have examined temperature and mortality in Latin America. We conducted a nonlinear, distributed-lag, longitudinal analysis of daily ambient temperatures and mortality among 326 Latin American cities between 2002 and 2015. We observed 15,431,532 deaths among ≈2.9 billion person-years of risk. The excess death fraction of total deaths was 0.67% (95% confidence interval (CI) 0.58–0.74%) for heat-related deaths and 5.09% (95% CI 4.64–5.47%) for cold-related deaths. The relative risk of death was 1.057 (95% CI 1.046-1.067%) per 1 °C higher temperature during extreme heat and 1.034 (95% CI 1.028-1.040%) per 1 °C lower temperature during extreme cold. In Latin American cities, a substantial proportion of deaths is attributable to nonoptimal ambient temperatures. Marginal increases in observed hot temperatures are associated with steep increases in mortality risk. These risks were strongest among older adults and for cardiovascular and respiratory deaths.

Anthropogenic greenhouse gas emissions continue to accelerate the pace of global climate change, with eight of the nine hottest years between 1880 and 2019 occurring since 2010 (ref. ^1^). The process of urbanization has also contributed to an increase in human exposure to extreme heat^2^, in particular through the urban heat island effect^3, 4^. Ambient temperatures in urban cores, where residents concentrate, can far exceed temperatures in periurban areas, causing urban residents to be especially exposed to extreme heat^2^.

Exposure to extreme hot and cold ambient temperatures has been linked to excess morbidity and premature mortality through a range of physiological mechanisms^5^. The human body regulates exposure to extreme heat primarily through vasodilatation, in which heat is transferred from the muscles to the skin via redistributed blood flow and by secreting sweat, which removes body heat through evaporation. Under conditions of extreme heat stress, these thermoregulatory processes can lead to increased cardiac demand, dehydration and pulmonary stress^5^. Thermoregulation during exposure to cold is driven by vasoconstriction and thermogenesis. Exceeding thermoregulatory capacity under extremely cold conditions can lead to decreased cardiac output, hypotension and eventual organ failure^6,^^7^. Notably, at a population level, cold seasonal temperatures are associated with increased circulation of influenza viruses^8^. As such, nonoptimal temperatures have been linked to a range of cardiovascular and respiratory causes of death^9^.

Recent global analyses^10–12^ and multiple regional studies in North America^13,14^, Europe^15^ and the Western Pacific^16^ have reported substantial impacts of nonoptimal temperatures on mortality, observing notable variations in the temperature–mortality relationship

between and within world regions^12^. These regional differences are probably driven by variations in the urban heat island effect^4^, climate, geography, built environment, social structures and existing adaptive capacity^17^. Accordingly, region-specific analyses are critically needed to understand the most vulnerable subpopulations, and to inform regional and local policies, emergency response plans and climate adaptation efforts. The vast majority of regional analyses of temperature and mortality have focused on high-income countries or included only a small number of cities in the Global South^18^. This reflects a paucity of research on climate and health in low- and middle-income countries more generally^19^, which hampers efforts to protect health in areas with the greatest susceptibility to climate change.

Latin America is one of the most urbanized regions of the world20 and therefore has a large population at risk of urban heat exposure^2^. A few studies in Latin America have examined the relationship between temperature and mortality within a single city^21–^^24^ or a small number of cities^25^, but examinations of the impact of ambient temperatures on health at a multinational or regional level within Latin America are lacking. A rare exception is a 2021 global study by Zhao et al. that included data from 66 locations in Latin America and the Caribbean, with most observed cities located in two countries (Brazil and Peru)^10^. In the coming decades, Latin America is projected to experience a substantial increase in mean annual temperature^26^ and, critically, an astounding increase in the frequency of extreme heat events^27^. Between the late twentieth century and mid-twenty-first century, the frequency of extremely hot days (defined by 95th percentile daily mean temperature between 1961 and 1990) in South America’s largest cities is projected to increase by five to ten times under the mid-level, representative concentration pathway 4.5 climate scenario^27^. A 2017 study, which included 32 locations in Mexico, Brazil and Chile, projected that, under multiple climate-change scenarios, midcentury decreases in cold-related mortality would approximately counterbalance increases in heat-related mortality, yet by the end of the twenty-first century overwhelming heat-related mortality would cause a substantial net increase in temperature-related excess mortality^28^. Current and future public health threats from these climatic changes are exacerbated by Latin America’s rapidly ageing population^29^, because advanced age is a known risk factor for temperature-related mortality^30^. Although Latin America has a similar proportion of individuals aged 65+ years (9.0% in 2020) compared with the global population (9.3%), this proportion of older adults is projected to increase more rapidly in Latin America (19.0% in 2050) than in the global population (15.9%)^29^. These intertwined challenges of increasing population exposure (urbanization), population susceptibility (aging population) and a warming climate make extreme temperatures a critical twenty-first-century environmental health challenge.

To better understand the relationship between temperature and mortality and to inform current and future efforts to prevent temperature-related deaths in Latin America, the present study characterized the impact of nonoptimal temperatures on all-cause and cause-specific mortality across 326 cities in 9 countries across Latin America.

## Results

### City characteristics

**Fig. 1:**
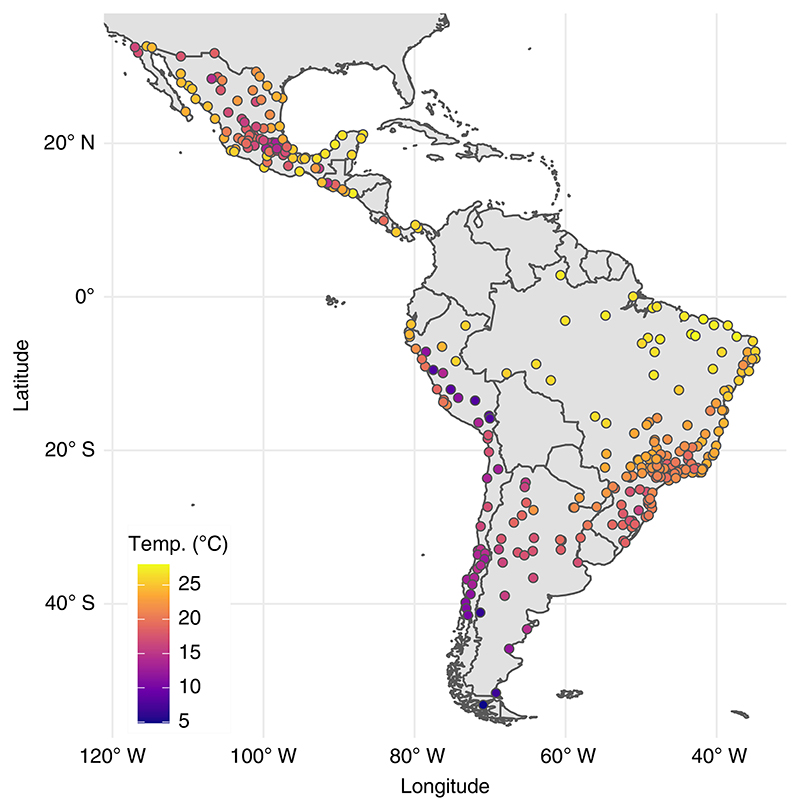
Annual mean temperatures during the city-specific observation period in 326 Latin American cities.

Overall and country-stratified characteristics of study cities are presented in Table 1. The median population among all cities was approximately 267,000 residents and 10% of cities had populations >1.2 million. The proportion of the population aged ≥65 years varied between countries, with a median of 5.3% aged 65+years among Peruvian cities and a median of 8.8% aged 65+ years among cities in Chile. The median city had 1,454 deaths per year during the study period (10th percentile 715 deaths, 90th percentile 6,825 deaths) and a grand total of 15,431,532 deaths was included in the analysis. These deaths occurred over ≈2.9 billion person-years of risk observed in the analysis. City names, average annual deaths and temperature summaries (median, 5th and 95th percentile) for each city are provided as Supplementary Table 1 and via an online interactive app (https://drexel-uhc.shinyapps.io/MS85). The locations and annual mean temperatures of all cities are presented in Fig. 1. In certain areas of Peru, Mexico and elsewhere, large differences in mean temperatures are apparent in cities that are relatively close together geographically. These variations are concentrated in mountainous regions with widely varying altitudes, which drives these temperature differences.

### Association between temperature and all-cause mortality

**Fig. 2:**
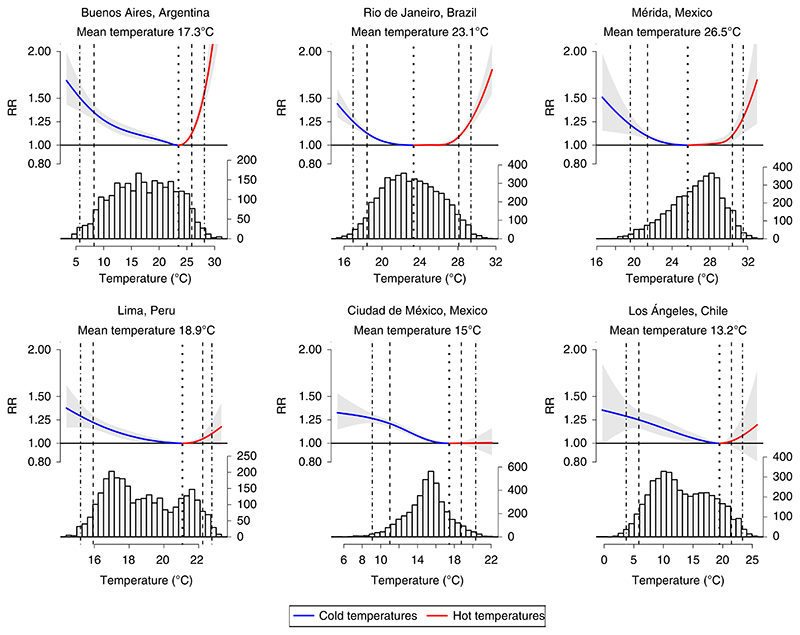
The city-specific temperature-mortality, exposure-response association (accumulated over 21 d) and distribution of daily temperatures for six selected cities. The blue and red solid lines represent temperature-mortality associations above (blue lines) and below (red lines) the minimum mortality temperature. Gray error bars represent 95% CIs. Vertical lines are placed at the optimal (that is, minimum mortality) temperature (dotted), the 5th and 95th percentiles of the temperature distribution (dashed) and 1st and 99th temperature percentiles (dash–dot).

Figure 2 shows estimated city-specific temperature–mortality associations alongside histograms of the daily temperature distribution for six cities selected to illustrate between-city differences in the temperature distributions and the shape of the temperature– mortality associations. Similar plots for all cities are presented in Supplementary Fig. 1. Most cities had an approximately *U*-shaped relationship between temperature and mortality, although the relationship between temperature and mortality was not symmetrical on both sides of the optimal temperature. At temperatures below the optimal temperature, mortality increased gradually as temperatures dropped, whereas at temperatures above the optimal temperature mortality increased more steeply as temperatures rose. The sharp increase in mortality with increasing temperatures was most pronounced for cities that regularly exceed approximately 25 °C (for example, Buenos Aires, Mérida and Rio de Janeiro in Fig. 2). However, among cities with temperate or cold climates that rarely (or never) exceed approximately 25 °C (for example, Lima, Mexico City and Los Angeles in Fig. 2), mortality did not increase or increased only minimally as temperatures increased.

**Fig. 3:**
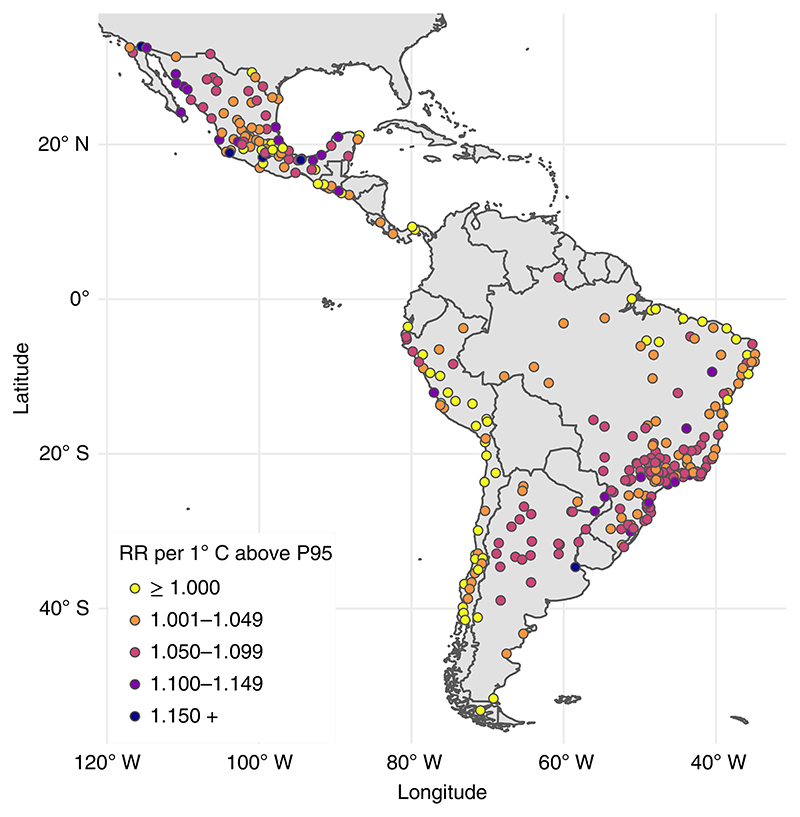
City-specific RR of heat-related mortality per 1 °C increase above the 95th percentile (P95) observed daily temperature in 326 Latin American cities.

The overall relative risk (RR) of all-cause mortality (all ages) at the city-specific 95th percentile relative to the minimum mortality temperature was 1.057 (95% CI 1.049–1.067). The overall RR of all-cause mortality (all ages) per 1 °C increase in daily mean temperature for temperatures above the city-specific 95th percentile was 1.057 (95% CI 1.046–1.067). However, this estimate varied geographically (Fig. 3). We observed a higher density of cities with larger increases in mortality per 1 °C higher temperature under extreme heat (for example, red and purple dots, representing RR ≥ 1.050) in relatively temperate areas of southern Brazil, Argentina and parts of Mexico. We found a higher density of cities with minimal changes in mortality under city-specific extreme heat (for example, yellow dots, representing RR ≤ 1.000) among cities in the high-altitude Andes (relatively cold) and northeast Brazil (tropical with relatively low temperature variability).

**Extended Data Fig. 1:**
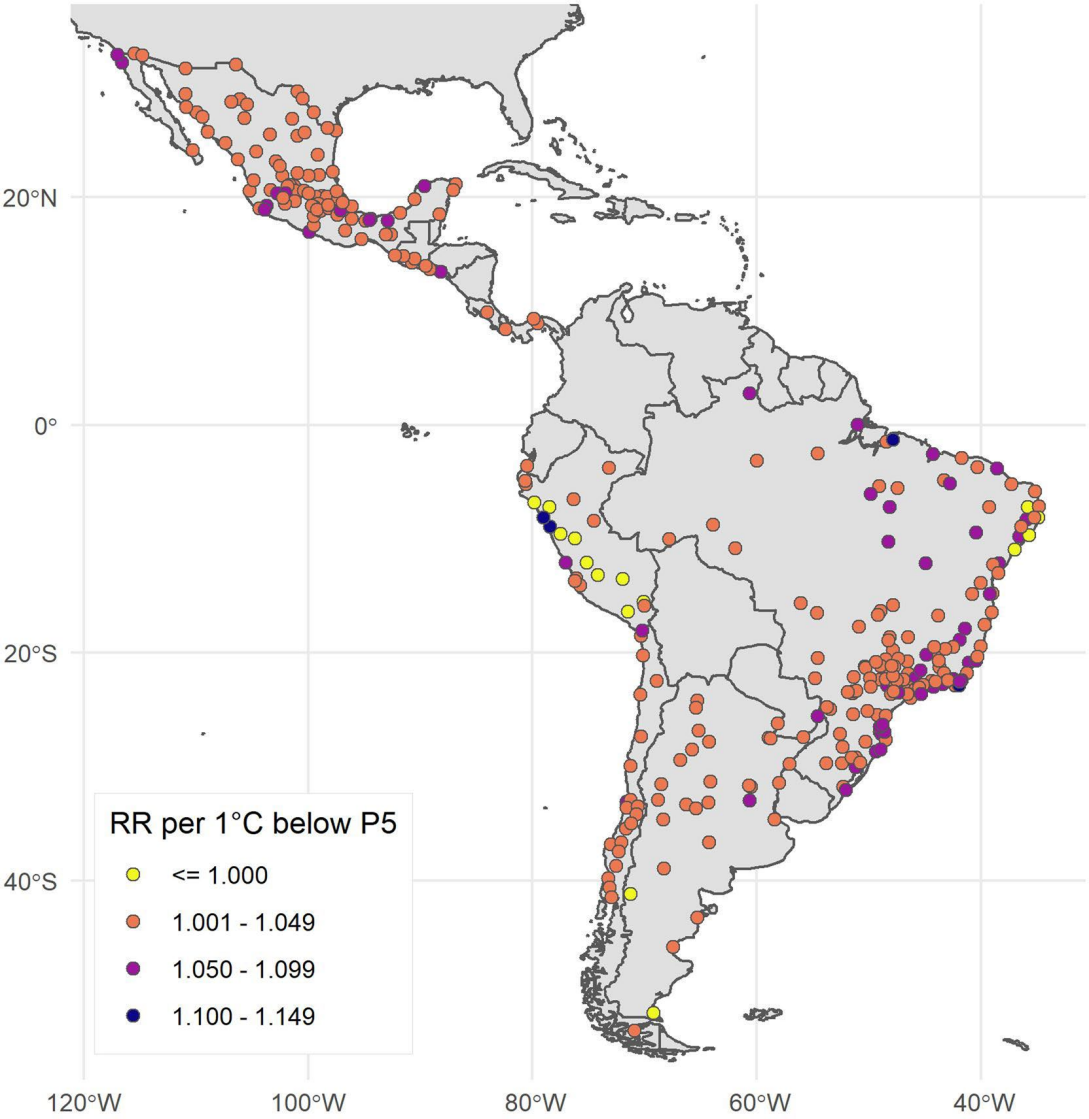
City-specific change in relative risk of mortality per 1 °C decrease in temperature below 5th percentile observed daily temperature. n/a.

For cold temperatures, the overall RR of all-cause mortality (all ages) at the city-specific 5th percentile relative to the minimum mortality temperature was 1.192 (95% CI 1.173–1.211). The RR of mortality per 1 °C decrease in extreme cold temperatures (<5th percentile) was 1.034 (95% CI 1.028–1.040). A map of the city-specific RR per 1 °C decrease during extreme cold (temperature below the 5th percentile) is presented in Extended Data Fig. 1.

Estimates of excess deaths from nonoptimal temperatures are presented in Table 2. We estimate that 5.75% (95% CI 5.31–6.07%) of deaths at all ages from all causes are associated with nonoptimal temperatures. The excess death fraction (EDF) for heat (the cumulative effect of all temperatures above optimal) was 0.67% (95% CI 0.58–0.74%), with extreme heat (≥95th percentile, city-specific observed temperatures) contributing a substantial portion of the heat-related excess deaths (0.42%, 95% CI 0.38–0.45%). The EDF from cold was substantially higher than heat, at 5.09% (95% CI 4.66–5.42%) for all cold and 1.03% (95% CI 0.99–1.06%) for extreme cold (≤5th percentile, city-specific observed temperatures). The EDF from nonoptimal temperatures was consistently higher among individuals aged 65+ years than among the total population (Table 2). City-specific minimum mortality temperature and EDFs for cold and heat among all ages are presented in Supplementary Table 1.

### Associations between temperature and cause-specific mortality

Compared with deaths from all causes and at all ages, we observed a substantially higher EDF from nonoptimal temperatures for cardiovascular disease (9.12% (95% CI 8.48, 9.70%)), respiratory diseases (10.73% (9.78, 11.50%)) and respiratory infections (12.09% (11.16, 12.82%)) (Table 2). The same pattern (higher EDFs for cardiovascular and respiratory deaths than for all-cause mortality) was observed for cold temperatures. In the case of hot temperatures, EDFs for respiratory mortality were slightly higher than for all-cause mortality, but EDFs for cardiovascular mortality were similar to those observed for all-cause mortality. Patterns were similar for deaths at all ages and >65 years, although the EDFs for >65 years were slightly higher than those for all ages.

**Extended Data Fig. 2:**
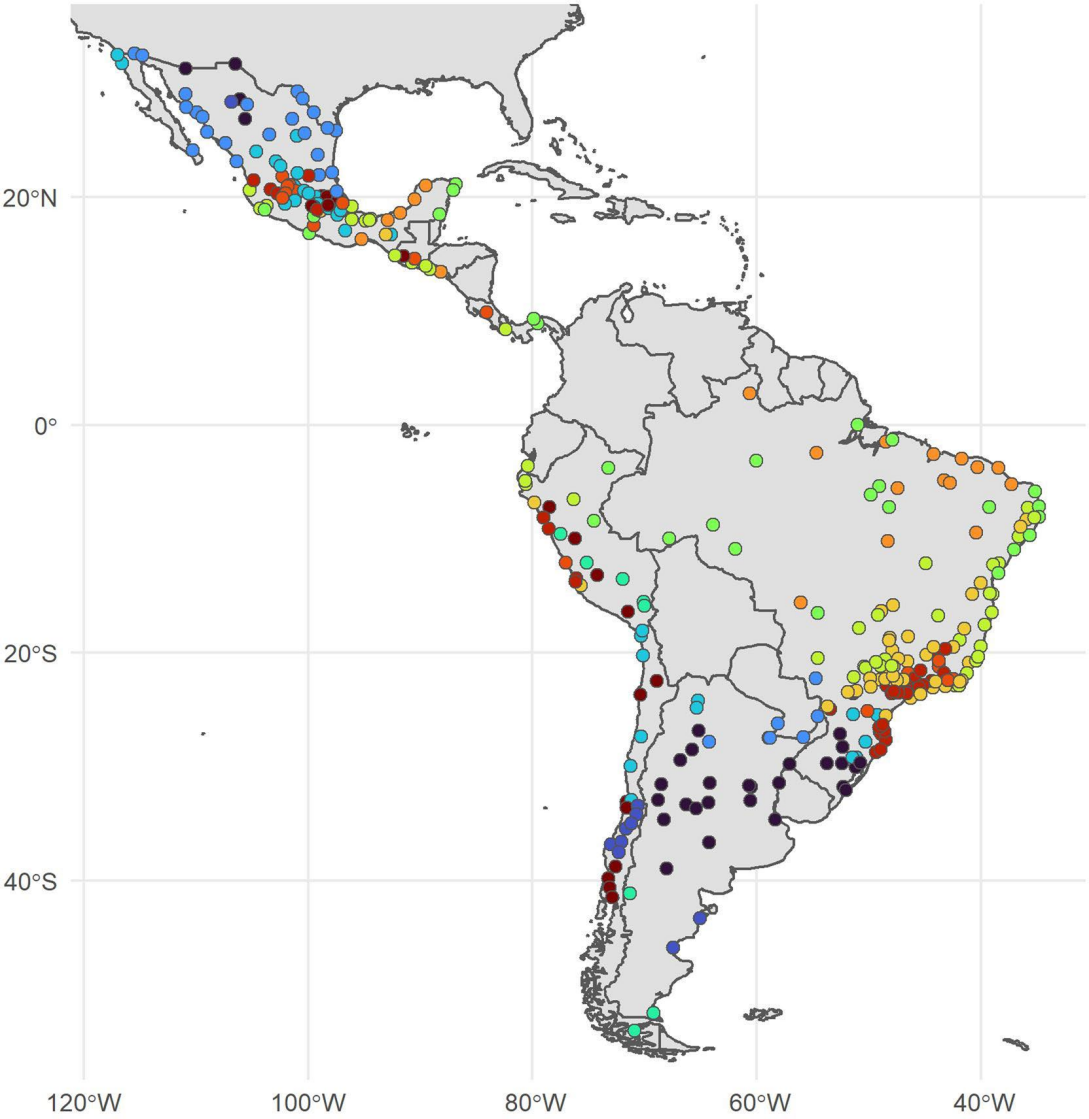
Cluster groupings in analyses of cause-specific mortality outcomes (by temperature, N = 12 clusters). n/a.

The cause of death most strongly associated with both heat and cold was respiratory infections, with 1.56% (95% CI 1.28–1.81%) of all-age deaths attributable to heat and 10.53% (95% CI 9.68–11.20%) to cold. A map of the city cluster groupings derived for the cause-specific analysis is presented in Extended Data Fig. 2.

## Discussion

We examined the contribution of ambient temperature to age- and cause-specific mortality across 326 cities in highly urbanized Latin America between 2002 and 2015. We found that a substantial proportion of mortality was attributable to ambient cold and to a lesser extent heat. This mortality burden is larger among older individuals and deaths from cardiovascular and respiratory causes. Importantly, we found that even small increases in extreme heat can rapidly increase mortality risk.

Overall, a substantially higher proportion of deaths is attributable to ambient cold than to ambient heat, which corroborates findings from similar analyses in other settings^9–12^. A 2021 analysis by Zhao et al. estimated temperature–mortality associations in 750 locations from 43 countries (including 66 locations in Latin America and the Caribbean), and extrapolated these estimates globally at 0.5° × 0.5° grid size (approximately 55 × 55 km^2^ at the equator) using meta-predictors^10^. The Zhao et al. study reported global EDFs of 8.52% for cold and 0.91% for heat for all-age, all-cause mortality. This global EDF for cold (8.52%) is almost twice our estimated EDF for cold within Latin American cities (4.71%). Although our study sample included some colder cities, this difference in mortality burden from cold temperatures may reflect the relatively temperate or warm climates within our study setting (median annual temp: 21.3 °C) compared with the Zhao et al. analysis (mean daily temperature: 15.2 °C), which includes densely populated regions in relatively colder climates in Europe, North America and the Western Pacific. Within Latin America and the Caribbean only, this same study estimated EDFs of 4.71% from cold and 1.06% from heat. This is quite similar to our estimated EDFs of 5.09% from cold and 0.67% from heat from observations in 326 Latin American cities. Differences in the Latin American regional estimates from Zhao et al. versus the present study may be explainable by Zhao et al. relying on larger cities to estimate temperature–mortality associations, which were then applied across the entire Latin American region (that is, urban and rural areas). In contrast, our study examines a wider range of city sizes (by population) and nearly five times more cities within the Latin American region, providing estimates for these cities without extrapolation to unobserved locations.

Drawing conclusions about the relative impact of heat and cold on mortality risk using EDFs is rendered complex by the fact that the EDF estimate is driven by both the impact of temperature changes on mortality and the distribution of hot and cold days. Our findings suggest that, even though mortality risk increases more gradually with decreasing temperatures below the optimal temperature than with increasing temperatures above the optimal temperature, the EDF attributable to cold is larger because there are generally more days below than above the optimal temperature. For this reason, we complemented the well-established methods for estimating temperature-related EDFs by calculating the difference in mortality risk per 1 °C increase under extreme temperatures above the city-specific 95th percentile (extreme heat) and per 1 °C decrease below the 5th percentile (extreme cold), representing the hottest and coldest 18 d within a typical year at each setting. Although mortality risk increased in a dose–response fashion both below and above optimal temperatures, the increase in mortality risk per 1 °C difference was notably steeper for extreme heat than for extreme cold temperatures (5.7% per 1 °C higher extreme heat (RR = 1.057) versus 3.4% per 1 °C lower extreme cold (RR = 1.034), respectively). These findings suggest that shifting temperature distributions to higher levels may at least initially result in pronounced increases in mortality risk as extreme heat becomes more frequent.

We also observed that the increase in mortality associated with a 1 °C increase in extremely hot temperatures has substantial geographic variation. For example, increases in mortality risk per 1 °C increase in extreme heat are particularly steep in the cities of coastal Mexico, northern Argentina and southern Brazil. Residents of these areas may be particularly vulnerable to extreme heat now and in the near term under even marginal increases in the frequency of extreme heat from climate change. Population-level adaptation to extreme temperatures is a complex mixture of individual factors (for example, clothing, underlying health), access to medical treatment for temperature-related morbidity and planned interventions to reduce exposures or medical vulnerability during extreme temperature events (for example, improved building design, public heating/cooling centers, emergency warning systems)^17^. A greater understanding of the city-level factors (physical, social or policy characteristics) that explain heterogeneity in temperature-related mortality may help identify effective actions to buffer future impacts of climatic changes.

Our results support the prevailing understanding that individuals aged ≥65 years have increased vulnerability to heat-related mortality^23,30^ and further emphasize that older individuals require prioritization within efforts to protect the public from extreme ambient temperatures. Compared with all-cause mortality, we found higher proportions of temperature-related deaths caused by cardiovascular diseases, noncommunicable respiratory diseases and respiratory infections. The proportion of deaths attributable to both heat and cold were higher for respiratory deaths than for all deaths combined. We found similar heat-related EDFs for cardiovascular deaths (0.69% (95% CI 0.64–0.74%)) and deaths from all causes (0.67% (95% CI 0.58–0.74%)), in contrast to other findings that cardiovascular mortality is a specific risk from heat exposure^9^. This could be related to regional reporting differences in causes of death as well as the lower prevalence of cardiovascular disease in Latin America (5,189 per 100,000 population) compared with global prevalence (6,762 per 100,000 population)^31^. Further analyses with more refined measures of the cause of death may shed light on the reasons for these results.

A key strength of the present study is the inclusion of all cities of ≥100,000 residents in 9 countries of Latin America, the world’s most urbanized region^20^ with a wide diversity of climates, populations and economic resources. We used two-phase statistical methods that allow us to capture local specificities while also permitting the pooling of information across multiple cities to derive more valid and reliable estimates of the impact of heat on mortality. An important byproduct of our approach is the generation of city-specific temperature–mortality curves, as well as the extension of this analysis to summarize the city-specific and overall changes in risks with marginal increases in extreme temperatures. The present study also used population-weighted, daily temperature reanalysis, which provides more spatially resolved estimates of true population exposure compared with the more common approach of applying measurements from one or a few temperature monitors to an entire city population^32^. Furthermore, we used individual-level mortality data, allowing us to examine variation in mortality by age and cause of death within a large number of cities in the Global South.

Our study has some limitations. Our exposure measure is limited because it does not account for interindividual differences in exposures. Physiological exposure to heat varies widely between individuals as a result of access to green vegetation, access to air-conditioning or other cooling mechanisms, characteristics of urban form and time–activity–location patterns^33^. The present paper is primarily descriptive and we were unable to account for potential inter- or intra-city ascertainment bias due to mortality-reporting practices or socioeconomic factors. Although available evidence does not suggest differences in heat-related mortality by sex^30^, it is important to expand our understanding of how climate change may impact sex differences in temperature exposures and vulnerability. We were unable to explore further the effect modification of age on the temperature–mortality relationship within more specific older age groupings due to the small numbers of daily deaths among older adults in many cities. Another limitation of our analysis is that we were not able to control for potential time-varying confounders, such as air pollution. The relationships between ambient temperature and specific air pollutants are complex, and there is some evidence of a two-way effect modification of the health impacts of air pollution and temperature that could influence our results^34,35^. These modifying effects could have differential impacts on our estimates of heat-versus cold-related mortality due to seasonal variations in concentrations of different air pollutants, which may also vary by location^36^. However, adjusting for air pollution as a confounder of the relationship between temperature and mortality may not be appropriate, because air pollution has no meaningful causal effect on ambient temperature and may instead serve as a causal intermediate in the relationship between temperature and population mortalit^37,38^. Finally, due to concerns about consistency in cause of death records in this large multi-country study, we were unable to look at more specific causes of death (for example, drowning) that have also been associated with nonoptimal temperatures^9^, and instead we rely on broader causes of death groupings.

Within a large and diverse sample of cities in the most urbanized region of the Global South, a substantial proportion of deaths can be attributed to ambient temperatures. These temperature-related deaths are particularly concentrated in older populations and are more common for deaths from cardiorespiratory diseases. Moreover, we observed that marginal increases in context-specific hot temperatures dramatically increase the risk of mortality. Although during the study period (2002–2015) cold temperatures contribute to more deaths than warm temperatures, our analysis affirms that the projected climate-related increases in the frequency of extremely hot days would probably substantially increase the risk of heat-related deaths across the region.

Although precise temperature-modeling estimates are needed to derive quantitative estimates of expected changes in deaths linked to global warming under different scenarios, our results suggest that rising temperatures will increase mortality above optimal temperatures by shifting more days to temperatures above the optimal temperature generally, but especially by shifting more days to levels >95th percentile (extreme heat), where heat-related deaths increase rapidly with increases in temperature. Policy-makers in Latin America and elsewhere must prioritize actions to prevent present and future health risks of extreme temperatures.

## Methods

### Study area

The present study was conducted as part of the Salud Urbana en América Latina (SALURBAL) project. The SALURBAL study protocol was approved by the Drexel University Institutional Review Board (ID no. 1612005035). The SALURBAL project has compiled and harmonized data on environmental, social and health characteristics for all cities of ≥100,000 residents (a total of 371 cities) in 11 Latin American countries^39^. Cities in SALURBAL were defined as urban agglomerations that contained >100,000 residents as of 2010 (ref. ^40^), allowing for examination of a range of city sizes from small cities to megacities. These cities are composed of clusters of administrative units encompassing the visually apparent urban built-up area as identified using satellite imagery^40^. In this analysis, we include 326 cities in Argentina, Brazil, Chile, Costa Rica, El Salvador, Guatemala, Mexico, Panama and Peru. Cities in Colombia and Nicaragua were excluded due to the limited availability of daily mortality data.

### Data sources

We used the ERA5-Land climate reanalysis with native ~9-km horizontal resolution^41^ to estimate the population-weighted daily mean ambient temperature for each city from 2002 to 2015. We used the reanalysis estimate of air temperature at 2 m above the land surface, which we refer to as ambient temperature, as a proxy for health-relevant environmental exposure. We calculated daily mean temperatures by averaging ERA5-Land hourly temperatures by calendar days. As ERA5-Land omits grid cells that contain >50% water, 99 of 326 cities (30%) contained ≥1 grid cells with missing temperature predictions within the city boundaries (mean percentage missing grid cells among 99 cities with ≥1 missing pixels: 18%). We imputed temperature values for missing grid cells using a random forest regression model that included resampled ERA5 temperature^42^ (31-km resolution), elevation and aspect (compass direction that terrain faces), with further modeling of the residuals using kriging spatial interpolation. To better approximate population exposures, we spatially weighted city temperature using 2010 estimates of the spatial distribution of the population (WorldPop, https://www.worldpop.org: Argentina, Brazil, Chile, Costa Rica, El Salvador, Guatemala and Mexico) or urban footprint (Global Urban Footprint, https://www.un-spider.org/node/11424: Panama and Peru).

Individual-level mortality data were compiled from vital registration systems in each country. Mortality records included date of death, municipality of residence, age at death and cause of death using the *International Classification of Diseases*, 10th revision^43^. We applied World Health Organization (WHO) Global Health Estimate (GHE) 2015 classifications^44^ to categorize deaths into the following groupings: all-cause (GHE tiers I, II, III), cardiovascular (II.G), noncommunicable respiratory disease (II.H) and respiratory infections (I.B). Due to concerns about accuracy and consistency in the cause of death records for diagnoses of specific diseases within this multi-country analysis, we limited the cause of death groupings to GHE second-tier categories. We selected these specific categories based on previous findings of strong associations between ambient temperature and mortality from cardiovascular diseases, noncommunicable respiratory diseases and respiratory infections^9^. We stratified deaths by age at death (<65 and 65+ years).

We compiled city-level population characteristics including total population and population age composition from census bureaus, national institutes of statistics or similar sources for each country^40^.

### Statistical analysis

We calculated descriptive summary statistics of city-level population, percentage of the population aged 65+ years, annual deaths and mean temperature, overall and stratified by country.

We estimated the temperature–mortality associations for deaths at all ages and stratified by age at death (<65 and 65+ years).

We modeled the nonlinear relationship between daily mean temperature and all-cause mortality using a two-stage approach^12,45^. First, for each city, we constructed a time series of daily all-cause and cause-specific mortality counts by aggregating individual-level mortality records within each city and age group (<65 and 65+ years) and linked it to the corresponding population-weighted daily mean temperature. We used distributed lag (0–21 d), nonlinear, conditional Poisson models to estimate city-specific, nonlinear associations between mortality and daily temperature with the logarithm of annual city population as an offset^45,46^. The nonlinear associations were estimated using natural cubic splines with knots placed at the minimum, maximum and 10th, 75th and 90th percentiles of the city-specific distribution of daily temperatures. We selected this number of knots and their locations based on their wide use in the previous literature^12^ and because they yielded equivalent or better model fit compared with others. The models were conditioned on strata defined by day of the week, month and year, offering strong control for seasonality and secular changes and yielding inferences based on short-term temperature variability. Second, we combined city-specific estimates using a random effects meta-regression to obtain smoothed (numerically stabilized) nonlinear association curves, given that some smaller cities have less precision due to their relatively few deaths. The dependent variables in the meta-regression were the four reduced spline coefficients that can be used to reconstruct the temperature–mortality association curve summed across lags^47^; the meta-predictors were each city’s median observed daily temperature, temperature range and country (Central American cities were treated as a single group due to the small number of study cities in each country).

The smoothed curves were used to estimate each city’s ‘optimal’ temperature, defined as the observed temperature where the temperature–mortality association curve achieved its minimum value^12^. The smoothed curves were displayed graphically for each city and, to communicate risk numerically, used to obtain two complementary types of summaries. First, we estimated EDFs from nonoptimal temperatures compared with the optimal temperature (also known as an attributable fraction). The EDF was defined as the ratio of estimated temperature-related excess deaths to total deaths for each city throughout the study period, expressed as a percentage. We estimated the EDF attributable to temperatures above or below the city-specific optimal temperature, as well as the EDF associated with either extreme heat or extreme cold, defined as ≥95th or ≤5th percentiles of city-specific daily temperatures, respectively. Second, for each city, we approximated the steepness or ‘slope’ of the nonlinear association curve under extreme heat (≥95th percentile temperature) and extreme cold (≤5th percentile), expressed as RR per 1 °C higher temperature. To obtain this ‘slope’ for extreme heat, we extracted the log(RR of mortality) at the 99th compared with the 95th percentile of the city-specific observed distribution of daily temperatures and divided it by the difference in °C between the 99th and 95th percentiles of the temperature distribution. We also estimated ‘slopes’ for extreme cold temperatures by dividing the log(RR at the 1st compared with the 5th percentile) of the city’s temperature distribution by the difference in °C between the 1st and 5th percentiles. Although the EDF has the advantage of combining information about the RR and the observed number of days above or below the optimal temperature, the second type of summary is useful in communicating risk in more intuitive units (that is, the difference in risk per °C higher temperature). We also report the pooled RR at the 5th and 95th percentiles of city-specific daily temperatures.

In addition, we estimated temperature–mortality associations with deaths from cause-specific groupings: cardiovascular disease, noncommunicable respiratory diseases and respiratory infections. Due to low counts of daily cause-specific deaths for many smaller cities, instead of city-specific analyses, we created 12 groups of cities with similar temperature distributions and estimated nonlinear temperature–mortality associations for each group. Cities were grouped via hierarchical clustering using Ward’s minimum variance method, with each city’s cumulative temperature distribution serving as input. Compared with clustering only on a handful of temperature summaries (for example, city’s temperature mean and variance), this approach groups cities based on the full temperature distribution. The same conditional Poisson models were used, with the addition of city identifier when forming strata on which to condition (that is, strata formed by city ID, year, month and day of the week).

All data collection was performed in R (v.3.6.0) and Python (v.3.9). All analyses were performed in R (v.3.6.0) and modeling was done with the mvmeta (v.1.0.3)^47^, dlnm (v.2.3.9)^48^ and gnm (v.1.1.1)^49^ packages. Clustering of cities was performed using the factoextra (v.1.0.7)^50^ package.

## Extended Data

Extended data are available for this paper at https://doi.org/10.1038/s41591-022-01872-6.

## Supplementary Material

Any methods, additional references, Nature Research reporting summaries, extended data, supplementary information, acknowledgements, peer review information; details of author contributions and competing interests; and statements of data and code availability are available at https://doi.org/10.1038/s41591-022-01872-6.

Fig. 1 and Table 1

Supplementary Infomration

## Figures and Tables

**Table 1:** Population, mortality and temperature characteristics of 326 Latin American cities

Countries in Latin America	No. of cities	Study period	City population (thousands)^a^	Percentage aged ≥65years^a^	Annual deaths^a^	Mean temperature (°C)^a^
All countries	326	2002–2015	267 (130, 1,292)	6.6 (4.6, 9.1)	1,454 (715, 6,825)	21.3 (14.9, 25.9)
Argentina	28	2009–2015	334 (133, 1,398)	8.2 (7.0, 12.0)	2,234 (821, 12,028)	17.5 (14.4, 21.6)
Brazil	152	2002–2015	221 (121, 1,292)	7.0 (4.8, 8.8)	1,443 (717, 6,279)	22.2 (18.9, 26.4)
Central America^b^	10	2009–2015	264 (185, 2,773)	7.2 (4.7, 8.4)	1,639 (1,085, 14,416)	23.8 (14.4, 25.8)
Chile	21	2004–2015	210 (140, 920)	8.8 (7.0, 10.7)	1,088 (771, 5,215)	13.7 (10.8, 17.0)
Mexico	92	2005–2015	352 (142, 1,089)	5.6 (4.4, 7.0)	1,825 (775, 5,272)	20.3 (15.6, 25.8)
Peru	23	2008–2015	288 (131, 885)	5.3 (3.9, 6.8)	978 (442, 3,644)	19.6 (8.0, 24.7)

a Median (10th, 90th percentiles).

b The Central America group in this analysis consists of cities in Guatemala, Panama, Costa Rica and El Salvador.

**Table 2:** Excess death fraction associated with nonoptimal temperatures

EDF	Total^a^	All heat	Extreme heat^b^	All cold	Extreme cold^c^
All-cause					
All ages (%)	5.75 (5.35, 6.12)	0.67 (0.58, 0.74)	0.42 (0.38, 0.45)	5.09 (4.64, 5.47)	1.03 (0.99, 1.06)
Ages 65+ (%)	7.63 (7.21, 7.97)	0.81 (0.75, 0.86)	0.55 (0.50, 0.59)	6.82 (6.41, 7.18)	1.36 (1.31, 1.39)
Cardiovascular					
All ages (%)	9.12 (8.48, 9.70)	0.69 (0.64, 0.74)	0.38 (0.36, 0.40)	8.43 (7.79, 9.01)	1.52 (1.48, 1.55)
Ages 65+ (%)	10.10 (9.17, 10.87)	0.75 (0.68, 0.82)	0.42 (0.38, 0.44)	9.35 (8.35, 10.13)	1.66 (1.59, 1.71)
Respiratory disease					
All ages (%)	10.73 (9.78, 11.50)	1.10 (1.02, 1.18)	0.54 (0.50, 0.57)	9.62 (8.55, 10.39)	1.58 (1.51, 1.63)
Ages 65+ (%)	10.59 (9.95, 11.07)	1.28 (1.13, 1.40)	0.61 (0.55, 0.65)	9.31 (8.71, 9.78)	1.64 (1.56, 1.69)
Respiratory infections					
All ages (%)	12.09 (11.16, 12.82)	1.56 (1.28, 1.81)	0.81 (0.77, 0.84)	10.53 (9.68, 11.20)	1.92 (1.86, 1.97)
Ages 65+ (%)	13.63 (12.15, 14.68)	1.84 (1.67, 1.95)	0.98 (0.92, 1.02)	11.79 (10.3, 12.80)	2.01 (1.90, 2.07)

a Percentage of total deaths explainable by temperatures above (‘All heat’) or below (‘All cold’) the city-specific optimal temperature.

b ≥95th percentile of the city-specific daily temperature distribution.

c ≤5th percentile of the city-specific daily temperature distribution.

## Data Availability

City-specific temperature and mortality summaries and analysis outputs are freely available from an interactive app at https://drexel-uhc.shinyapps.io/MS85. Links to the ERA5-Land, WorldPop and Global Urban Footprint source datasets used to estimate population-weighted ambient temperature, as well as final daily temperature outputs, are available at https://github.com/Drexel-UHC/salurbal_heat. Vital registration and population data for Brazil, Chile and Mexico were downloaded from publicly available repositories of statistical agencies in each country. Vital registration and population data for Argentina, Costa Rica, El Salvador, Guatemala, Panama and Peru were obtained directly from statistical agencies in each country. A link to these agency websites can be accessed via https://drexel.edu/lac/data-evidence/data-acknowledgements.

## Reporting summary

Further information on research design is available in the Nature Research Reporting Summary linked to this article.

## Code availability

The code repository for the estimation of city-specific daily temperature is available at https://github.com/Drexel-UHC/salurbal_heat. The code repository for the main statistical analysis is available at https://github.com/Drexel-UHC/SALURBAL-Heat-Study-MS85.
