# Supplementary material for: City-level impact of extreme temperatures and mortality in Latin America
Source: Nat Med. Author manuscript; Available in PMC 2022 Sep 8. (PMC9388372; doi:10.1038/s41591-022-01872-6)
Supplement: Fig. 1 and Table 1 [file EMS151808-supplement-Fig__1_and_Table_1.pdf]

nature portfolio

https://doi.org/10.1038/s41591-022-01872-6

Supplementary information

City-level impact of extreme temperatures and mortality in Latin America

In the format provided by the authors and unedited

Supplementary Information for:

City-level impact of extreme temperatures and mortality in Latin America

Authors: Josiah L. Kephart, Brisa N. Sánchez, Jeffrey Moore, Leah H. Schinasi, Maryia Bakhtsiyarava, Yang Ju, Nelson Gouveia, Waleska T Caiaffa, Iryna Dronova, Saravanan Arunachalam, Ana V. Diez Roux, Daniel A. Rodríguez

Corresponding Author:
Josiah L. Kephart, PhD, MPH
Email: jlk465@drexel.edu

Supplementary Figure 1. The city-specific temperature-mortality exposure-response association (accumulated over 21 days) and distribution of daily temperatures for 326 Latin American cities. The blue and red solid lines represent temperature-mortality associations above (blue lines) and below (red lines) the minimum mortality temperature. Grey error bars represent 95% confidence intervals. Vertical lines are placed at the optimal (i.e. minimum mortality) temperature (dotted), the 5th and 95th percentiles of the temperature distribution (dashed), and 1st and 99th temperature percentiles (dash-dot).

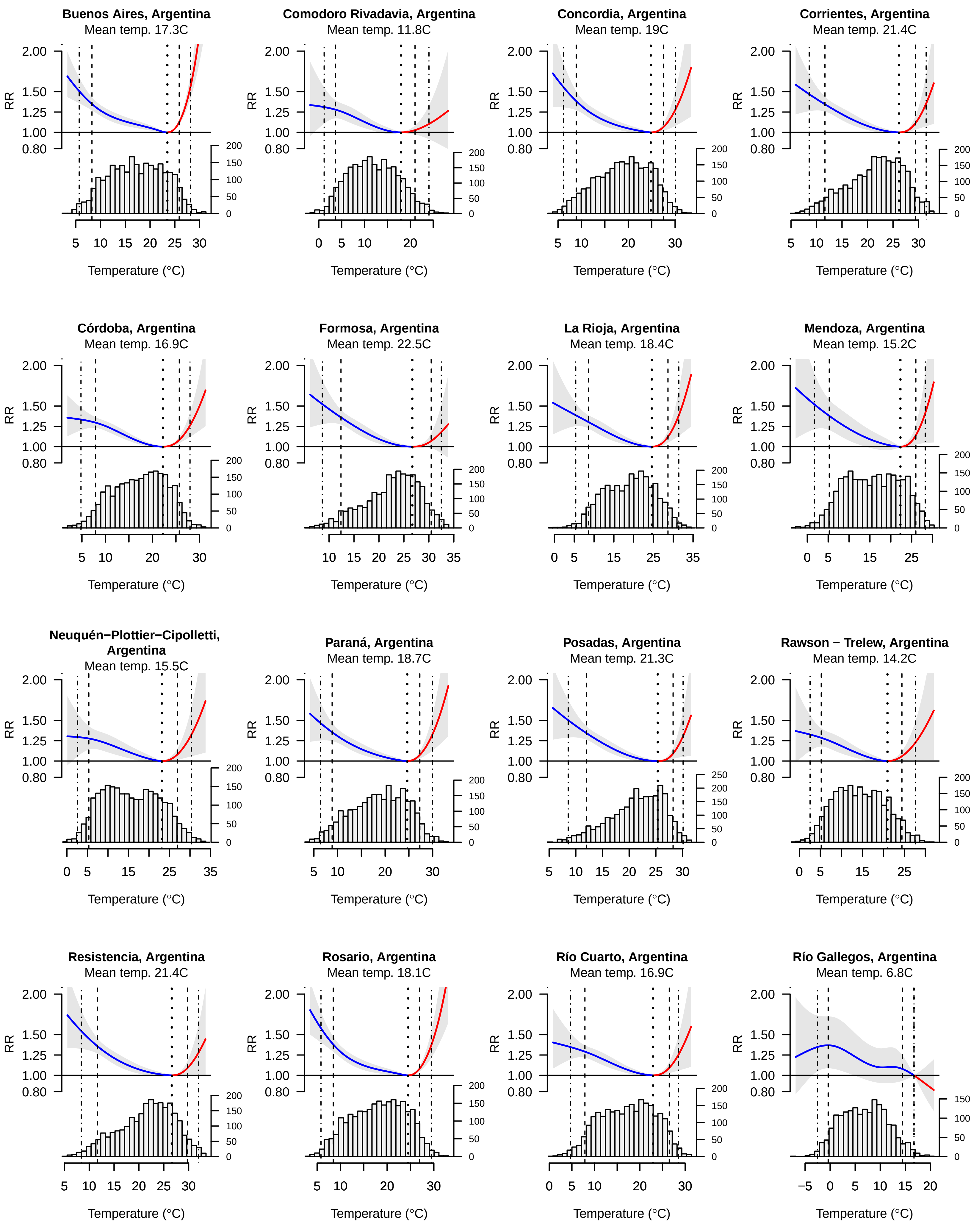

Buenos Aires, Argentina
Mean temp. 17.3C
Comodoro Rivadavia, Argentina
Mean temp. 11.8C
Concordia, Argentina
Mean temp. 19C
Corrientes, Argentina
Mean temp. 21.4C
Córdoba, Argentina
Mean temp. 16.9C
Formosa, Argentina
Mean temp. 22.5C
La Rioja, Argentina
Mean temp. 18.4C
Mendoza, Argentina
Mean temp. 15.2C
Neuquén−Plottier−Cipolletti, Argentina
Mean temp. 15.5C
Paraná, Argentina
Mean temp. 18.7C
Posadas, Argentina
Mean temp. 21.3C
Rawson − Trelew, Argentina
Mean temp. 14.2C
Resistencia, Argentina
Mean temp. 21.4C
Rosario, Argentina
Mean temp. 18.1C
Río Cuarto, Argentina
Mean temp. 16.9C
Río Gallegos, Argentina
Mean temp. 6.8C
RR
Temperature (°C)

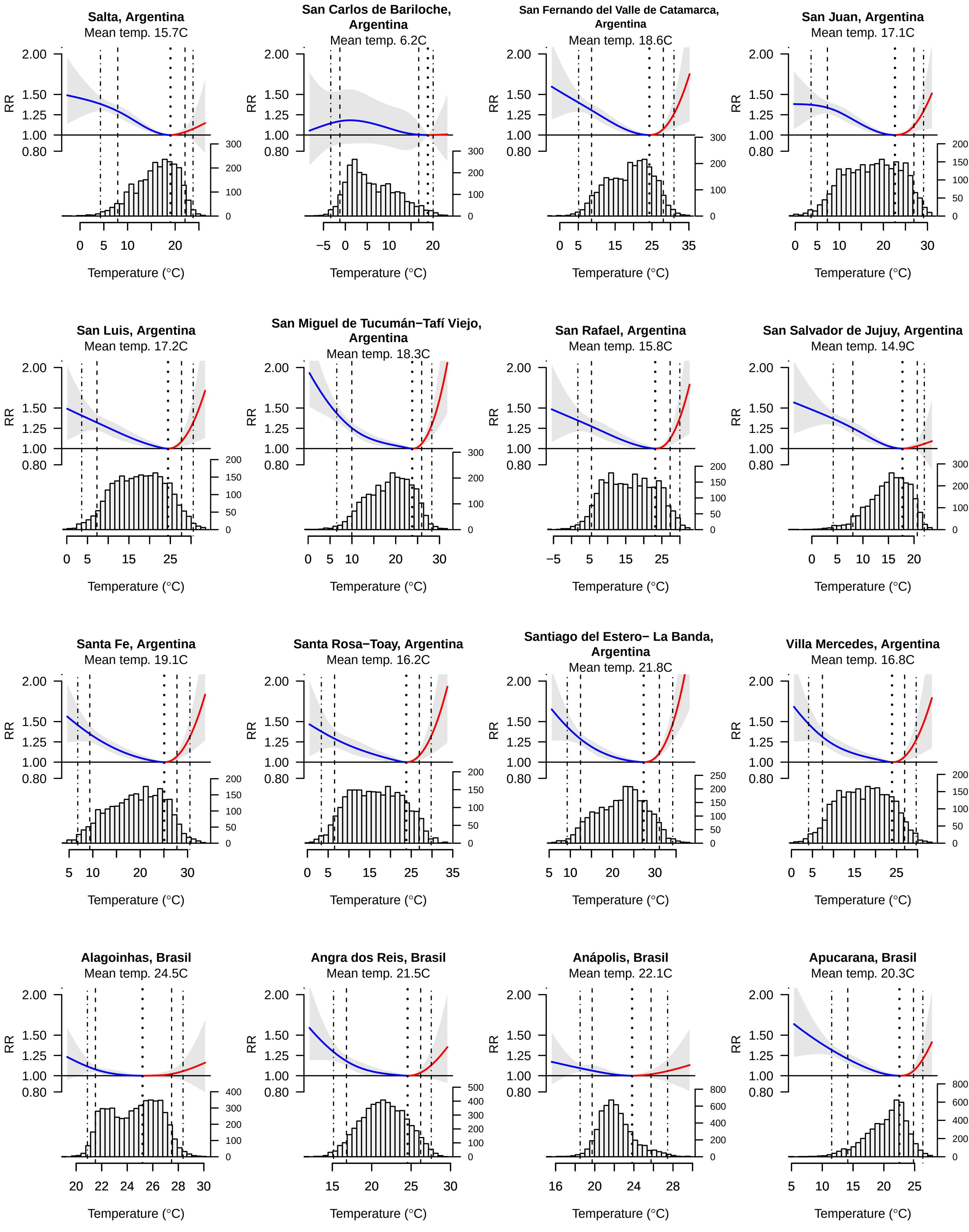
Salta, Argentina
Mean temp. 15.7C
San Carlos de Bariloche, Argentina
Mean temp. 6.2C
San Fernando del Valle de Catamarca, Argentina
Mean temp. 18.6C
San Juan, Argentina
Mean temp. 17.1C
San Luis, Argentina
Mean temp. 17.2C
San Miguel de Tucumán−Tafí Viejo, Argentina
Mean temp. 18.3C
San Rafael, Argentina
Mean temp. 15.8C
San Salvador de Jujuy, Argentina
Mean temp. 14.9C
Santa Fe, Argentina
Mean temp. 19.1C
Santa Rosa−Toay, Argentina
Mean temp. 16.2C
Santiago del Estero− La Banda, Argentina
Mean temp. 21.8C
Villa Mercedes, Argentina
Mean temp. 16.8C
Alagoinhas, Brasil
Mean temp. 24.5C
Angra dos Reis, Brasil
Mean temp. 21.5C
Anápolis, Brasil
Mean temp. 22.1C
Apucarana, Brasil
Mean temp. 20.3C
RR
Temperature (°C)

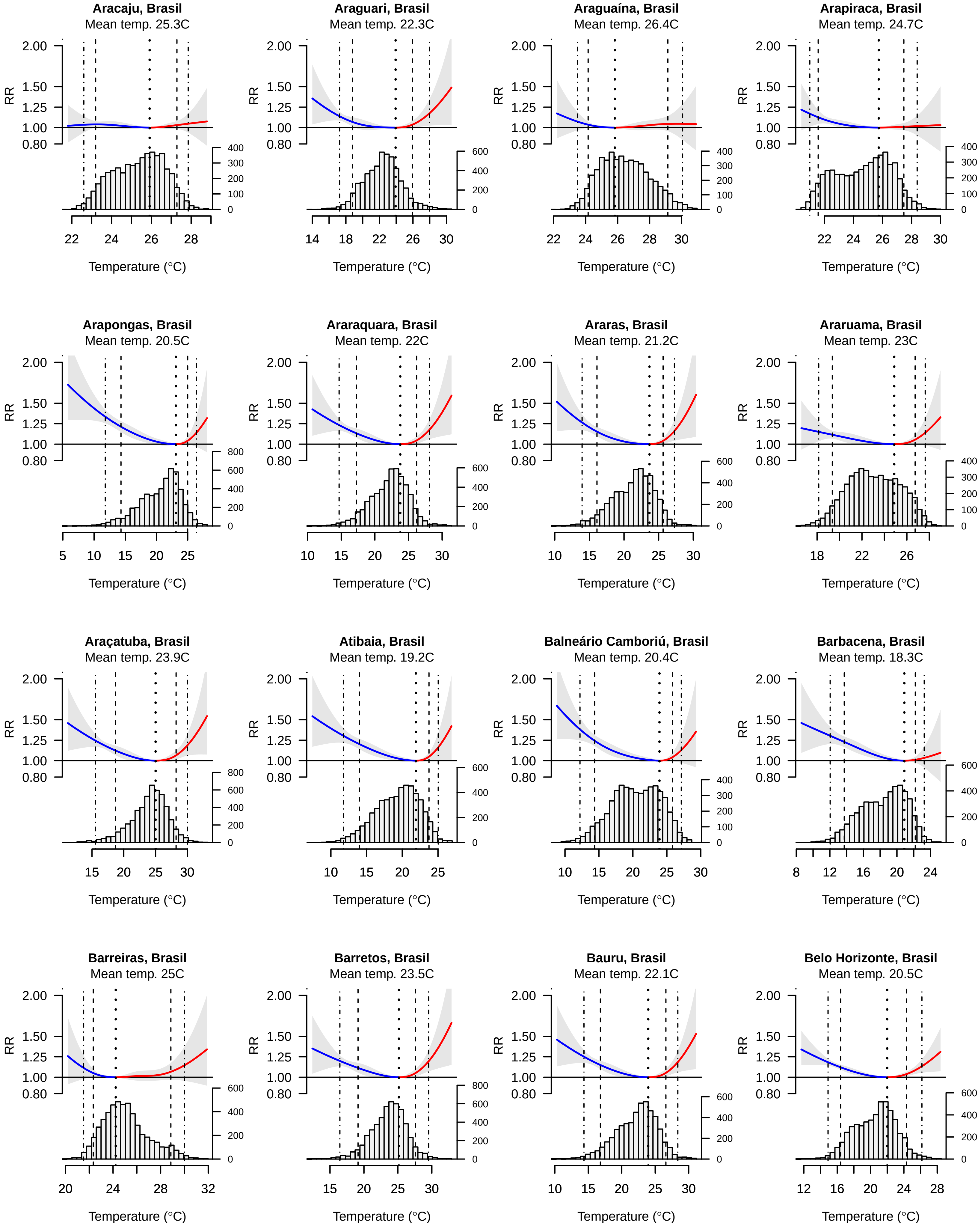
Aracaju, Brasil
Mean temp. 25.3C
Araguari, Brasil
Mean temp. 22.3C
Araguaína, Brasil
Mean temp. 26.4C
Arapiraca, Brasil
Mean temp. 24.7C
Arapongas, Brasil
Mean temp. 20.5C
Araraquara, Brasil
Mean temp. 22C
Araras, Brasil
Mean temp. 21.2C
Araruama, Brasil
Mean temp. 23C
Araçatuba, Brasil
Mean temp. 23.9C
Atibaia, Brasil
Mean temp. 19.2C
Balneário Camboriú, Brasil
Mean temp. 20.4C
Barbacena, Brasil
Mean temp. 18.3C
Barreiras, Brasil
Mean temp. 25C
Barretos, Brasil
Mean temp. 23.5C
Bauru, Brasil
Mean temp. 22.1C
Belo Horizonte, Brasil
Mean temp. 20.5C
RR
Temperature (°C)

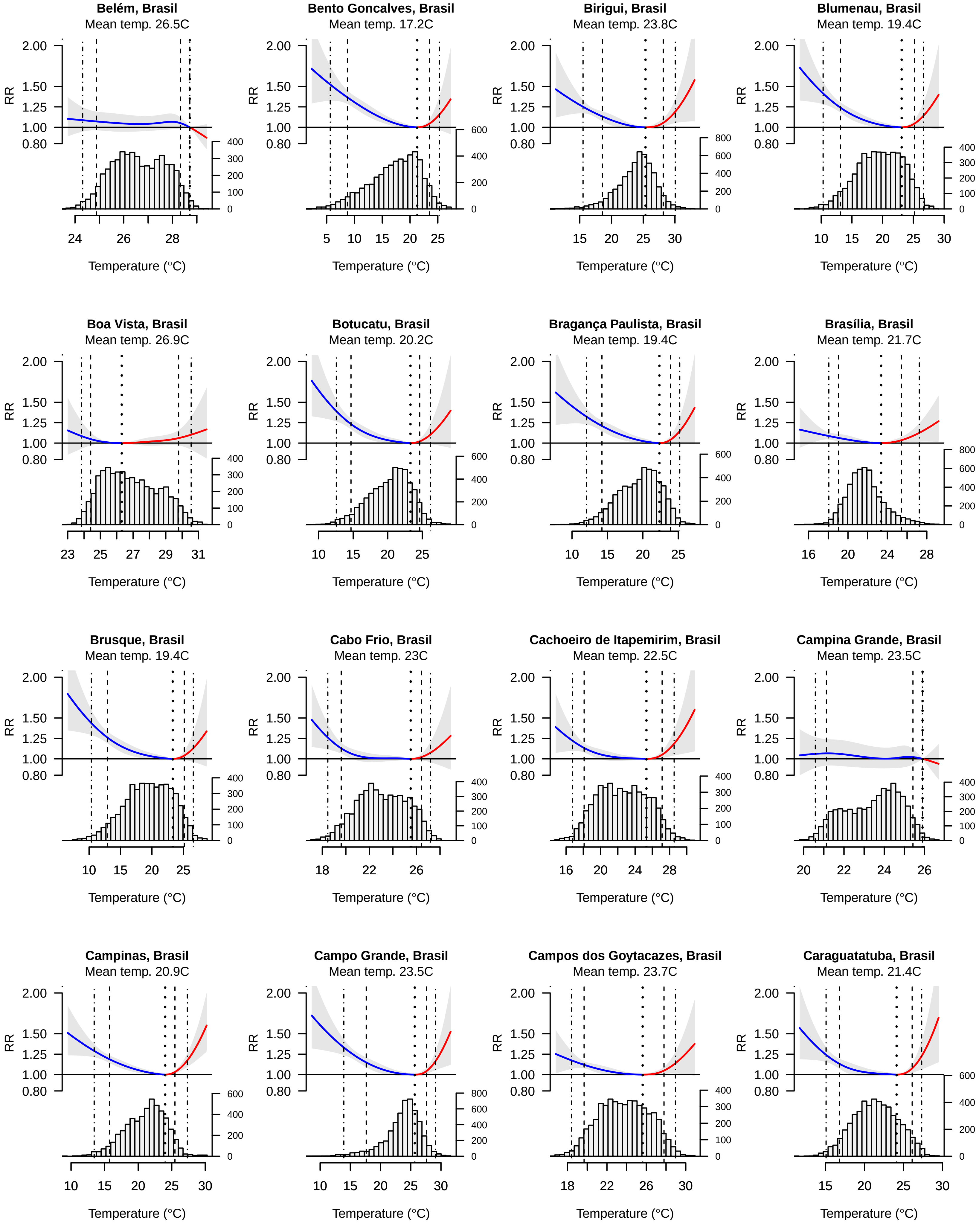
Belém, Brasil
Mean temp. 26.5C
Bento Goncalves, Brasil
Mean temp. 17.2C
Birigui, Brasil
Mean temp. 23.8C
Blumenau, Brasil
Mean temp. 19.4C
Boa Vista, Brasil
Mean temp. 26.9C
Botucatu, Brasil
Mean temp. 20.2C
Bragança Paulista, Brasil
Mean temp. 19.4C
Brasília, Brasil
Mean temp. 21.7C
Brusque, Brasil
Mean temp. 19.4C
Cabo Frio, Brasil
Mean temp. 23C
Cachoeiro de Itapemirim, Brasil
Mean temp. 22.5C
Campina Grande, Brasil
Mean temp. 23.5C
Campinas, Brasil
Mean temp. 20.9C
Campo Grande, Brasil
Mean temp. 23.5C
Campos dos Goytacazes, Brasil
Mean temp. 23.7C
Caraguatatuba, Brasil
Mean temp. 21.4C
RR
Temperature (°C)

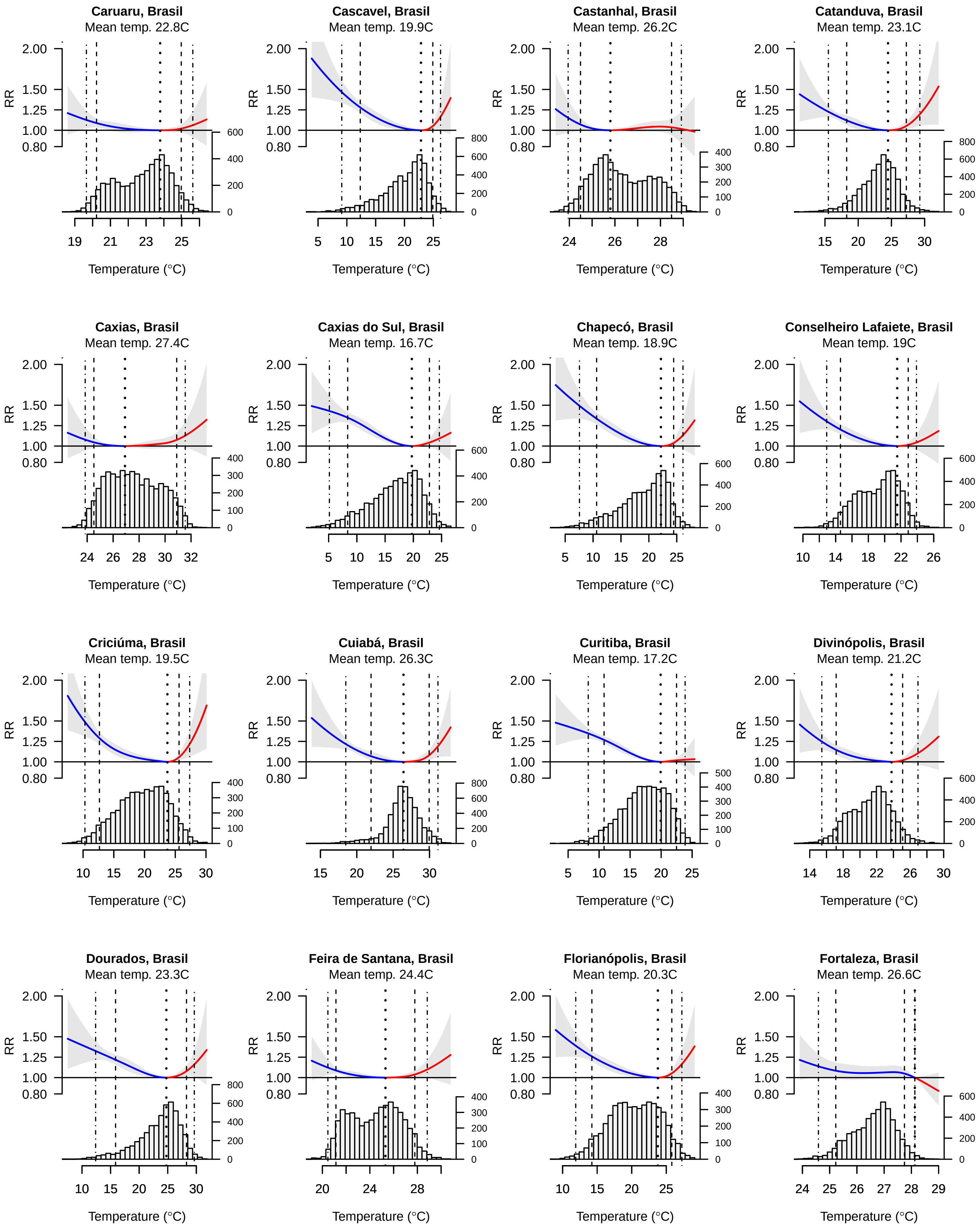

Caruaru, Brasil
Mean temp. 22.8C
Cascavel, Brasil
Mean temp. 19.9C
Castanhal, Brasil
Mean temp. 26.2C
Catanduva, Brasil
Mean temp. 23.1C
Caxias, Brasil
Mean temp. 27.4C
Caxias do Sul, Brasil
Mean temp. 16.7C
Chapecó, Brasil
Mean temp. 18.9C
Conselheiro Lafaiete, Brasil
Mean temp. 19C
Criciúma, Brasil
Mean temp. 19.5C
Cuiabá, Brasil
Mean temp. 26.3C
Curitiba, Brasil
Mean temp. 17.2C
Divinópolis, Brasil
Mean temp. 21.2C
Dourados, Brasil
Mean temp. 23.3C
Feira de Santana, Brasil
Mean temp. 24.4C
Florianópolis, Brasil
Mean temp. 20.3C
Fortaleza, Brasil
Mean temp. 26.6C
RR
Temperature (°C)

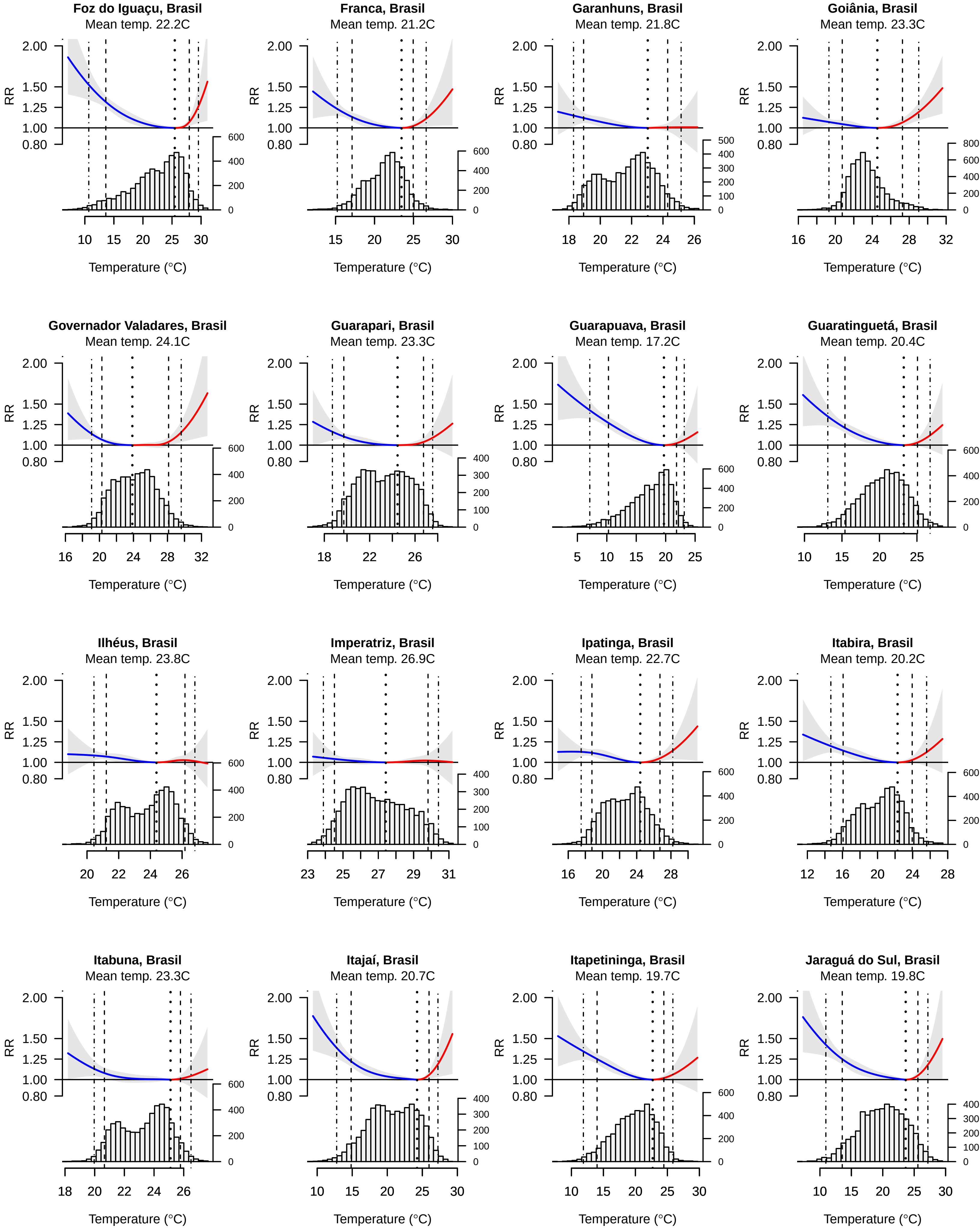
Foz do Iguaçu, Brasil
Mean temp. 22.2C
Franca, Brasil
Mean temp. 21.2C
Garanhuns, Brasil
Mean temp. 21.8C
Goiânia, Brasil
Mean temp. 23.3C
Governador Valadares, Brasil
Mean temp. 24.1C
Guarapari, Brasil
Mean temp. 23.3C
Guarapuava, Brasil
Mean temp. 17.2C
Guaratinguetá, Brasil
Mean temp. 20.4C
Ilhéus, Brasil
Mean temp. 23.8C
Imperatriz, Brasil
Mean temp. 26.9C
Ipatinga, Brasil
Mean temp. 22.7C
Itabira, Brasil
Mean temp. 20.2C
Itabuna, Brasil
Mean temp. 23.3C
Itajaí, Brasil
Mean temp. 20.7C
Itapetininga, Brasil
Mean temp. 19.7C
Jaraguá do Sul, Brasil
Mean temp. 19.8C
RR
Temperature (°C)

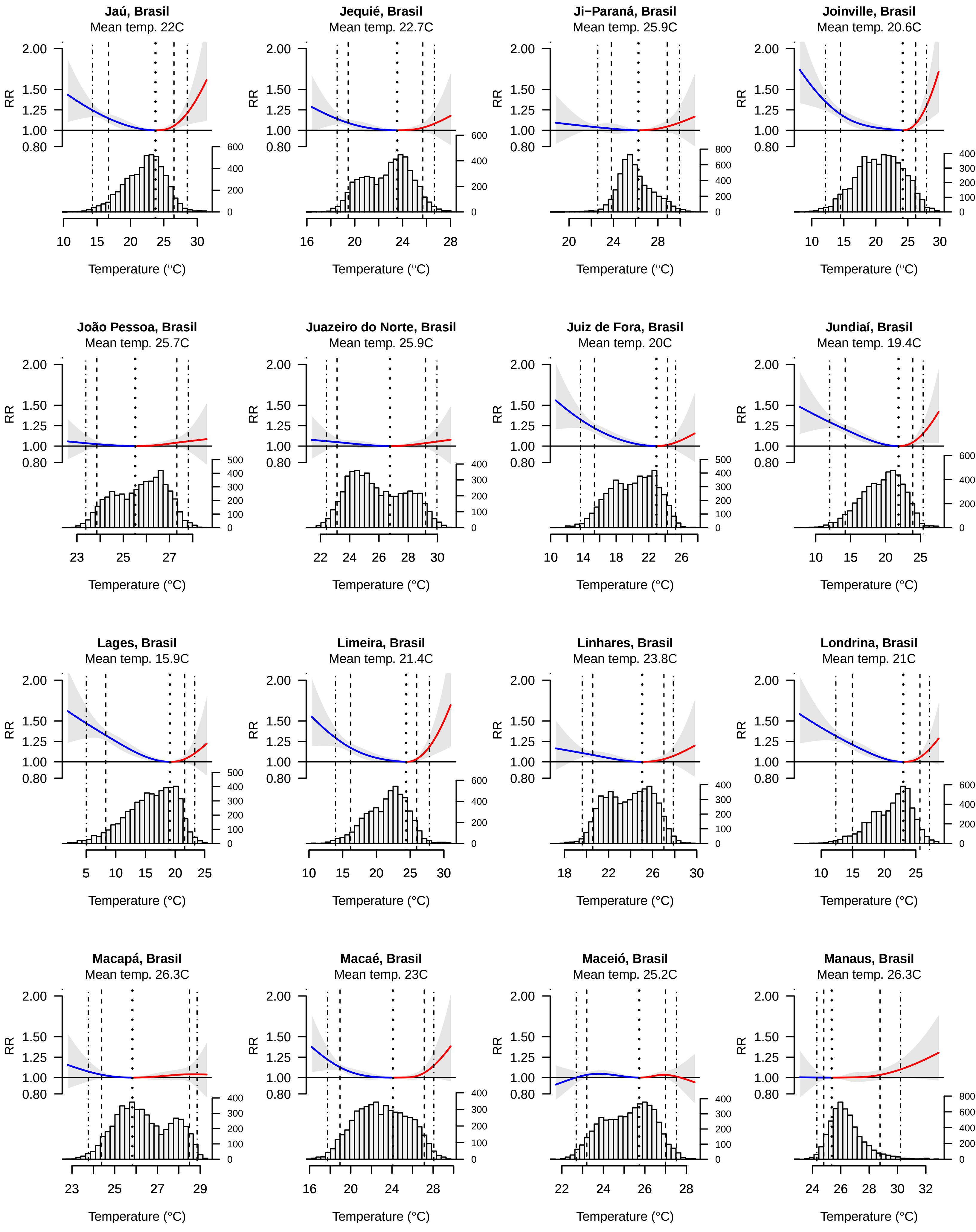

Jaú, Brasil
Mean temp. 22C
Jequié, Brasil
Mean temp. 22.7C
Ji−Paraná, Brasil
Mean temp. 25.9C
Joinville, Brasil
Mean temp. 20.6C
João Pessoa, Brasil
Mean temp. 25.7C
Juazeiro do Norte, Brasil
Mean temp. 25.9C
Juiz de Fora, Brasil
Mean temp. 20C
Jundiaí, Brasil
Mean temp. 19.4C
Lages, Brasil
Mean temp. 15.9C
Limeira, Brasil
Mean temp. 21.4C
Linhares, Brasil
Mean temp. 23.8C
Londrina, Brasil
Mean temp. 21C
Macapá, Brasil
Mean temp. 26.3C
Macaé, Brasil
Mean temp. 23C
Maceió, Brasil
Mean temp. 25.2C
Manaus, Brasil
Mean temp. 26.3C
RR
Temperature (°C)

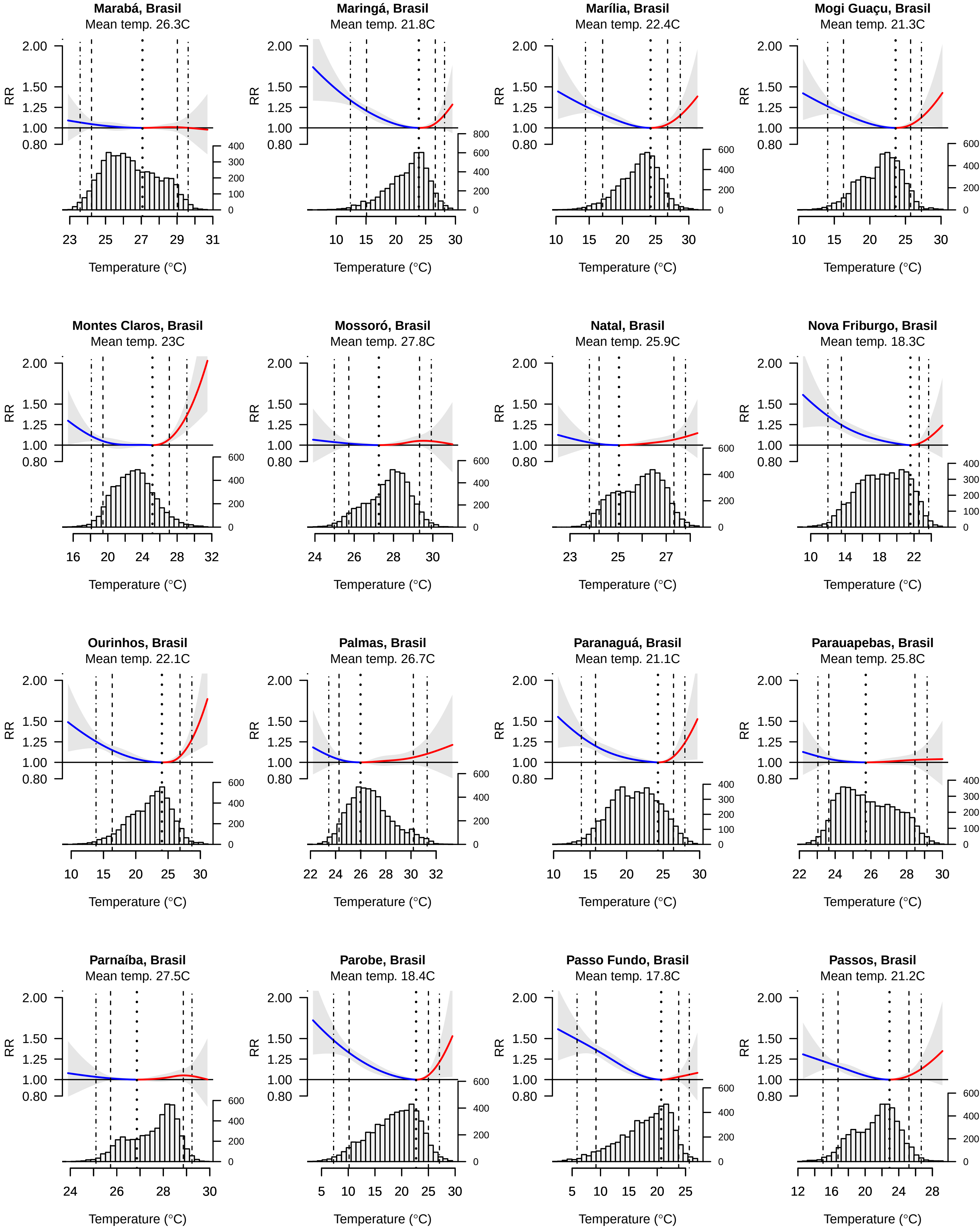
Marabá, Brasil
Mean temp. 26.3C
Maringá, Brasil
Mean temp. 21.8C
Marília, Brasil
Mean temp. 22.4C
Mogi Guaçu, Brasil
Mean temp. 21.3C
Montes Claros, Brasil
Mean temp. 23C
Mossoró, Brasil
Mean temp. 27.8C
Natal, Brasil
Mean temp. 25.9C
Nova Friburgo, Brasil
Mean temp. 18.3C
Ourinhos, Brasil
Mean temp. 22.1C
Palmas, Brasil
Mean temp. 26.7C
Paranaguá, Brasil
Mean temp. 21.1C
Parauapebas, Brasil
Mean temp. 25.8C
Parnaíba, Brasil
Mean temp. 27.5C
Parobe, Brasil
Mean temp. 18.4C
Passo Fundo, Brasil
Mean temp. 17.8C
Passos, Brasil
Mean temp. 21.2C
RR
Temperature (°C)

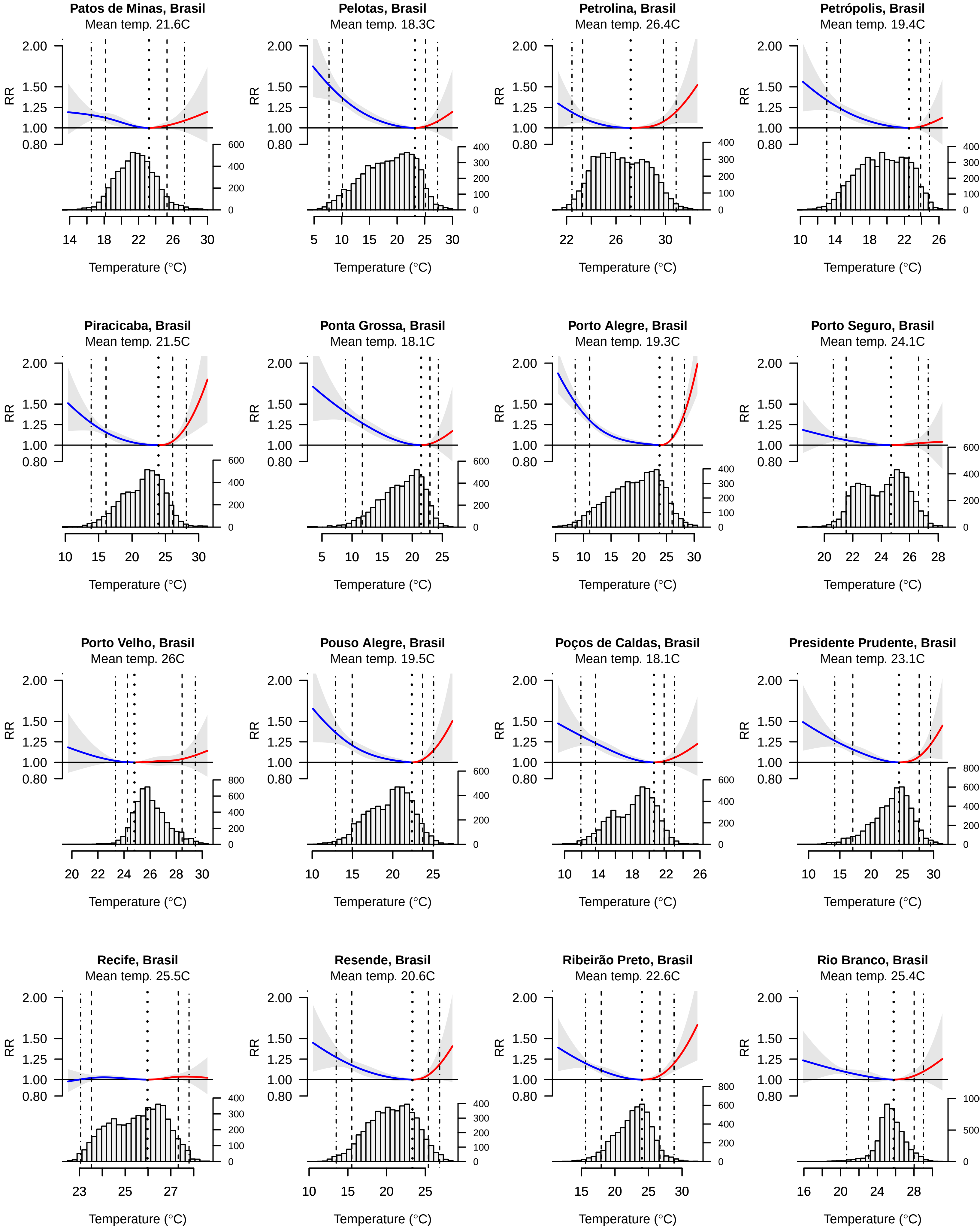

Patos de Minas, Brasil
Mean temp. 21.6C
Pelotas, Brasil
Mean temp. 18.3C
Petrolina, Brasil
Mean temp. 26.4C
Petrópolis, Brasil
Mean temp. 19.4C
Piracicaba, Brasil
Mean temp. 21.5C
Ponta Grossa, Brasil
Mean temp. 18.1C
Porto Alegre, Brasil
Mean temp. 19.3C
Porto Seguro, Brasil
Mean temp. 24.1C
Porto Velho, Brasil
Mean temp. 26C
Pouso Alegre, Brasil
Mean temp. 19.5C
Poços de Caldas, Brasil
Mean temp. 18.1C
Presidente Prudente, Brasil
Mean temp. 23.1C
Recife, Brasil
Mean temp. 25.5C
Resende, Brasil
Mean temp. 20.6C
Ribeirão Preto, Brasil
Mean temp. 22.6C
Rio Branco, Brasil
Mean temp. 25.4C
RR
Temperature (°C)

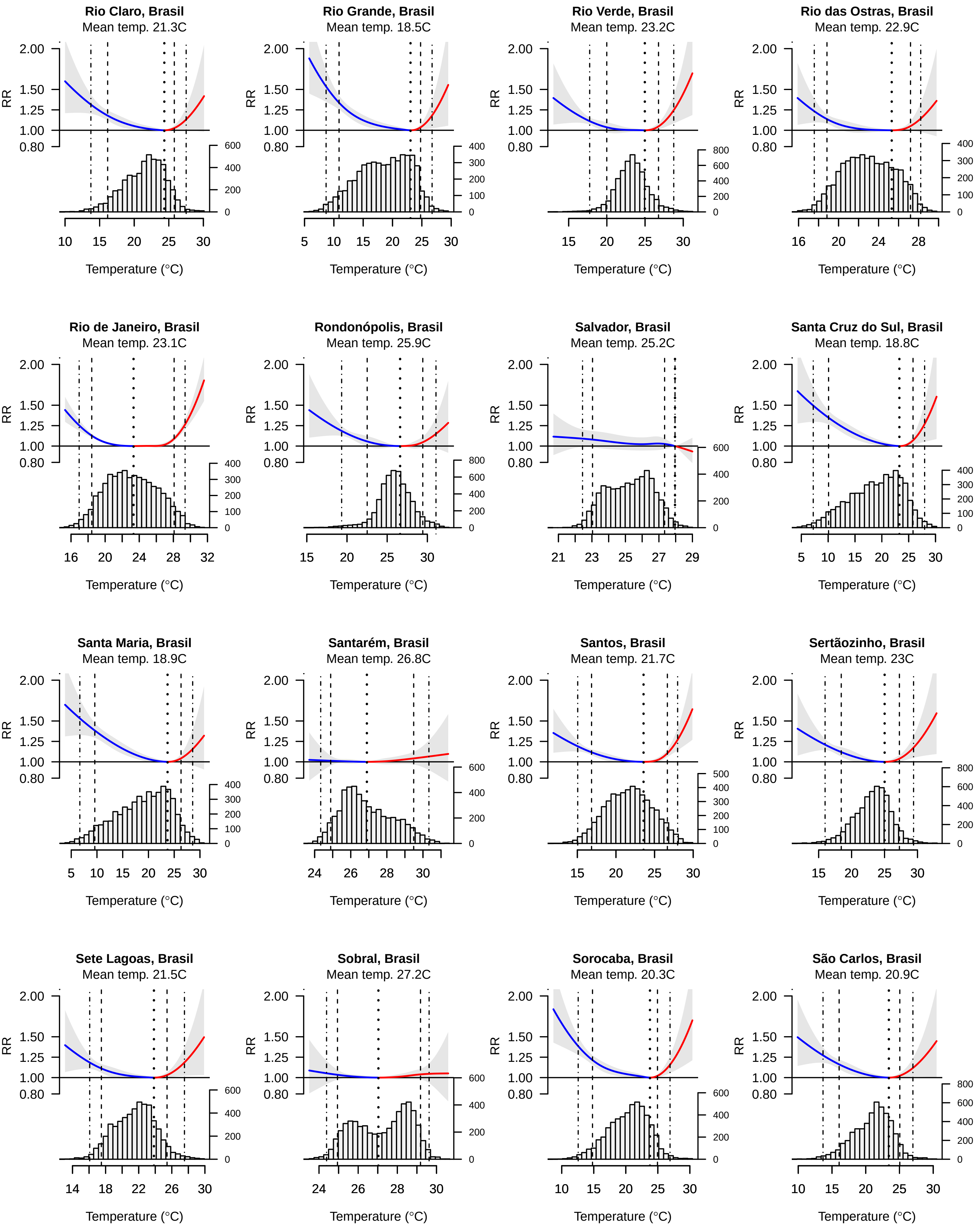
Rio Claro, Brasil
Mean temp. 21.3C
Rio Grande, Brasil
Mean temp. 18.5C
Rio Verde, Brasil
Mean temp. 23.2C
Rio das Ostras, Brasil
Mean temp. 22.9C
Rio de Janeiro, Brasil
Mean temp. 23.1C
Rondonópolis, Brasil
Mean temp. 25.9C
Salvador, Brasil
Mean temp. 25.2C
Santa Cruz do Sul, Brasil
Mean temp. 18.8C
Santa Maria, Brasil
Mean temp. 18.9C
Santarém, Brasil
Mean temp. 26.8C
Santos, Brasil
Mean temp. 21.7C
Sertãozinho, Brasil
Mean temp. 23C
Sete Lagoas, Brasil
Mean temp. 21.5C
Sobral, Brasil
Mean temp. 27.2C
Sorocaba, Brasil
Mean temp. 20.3C
São Carlos, Brasil
Mean temp. 20.9C
RR
Temperature (°C)

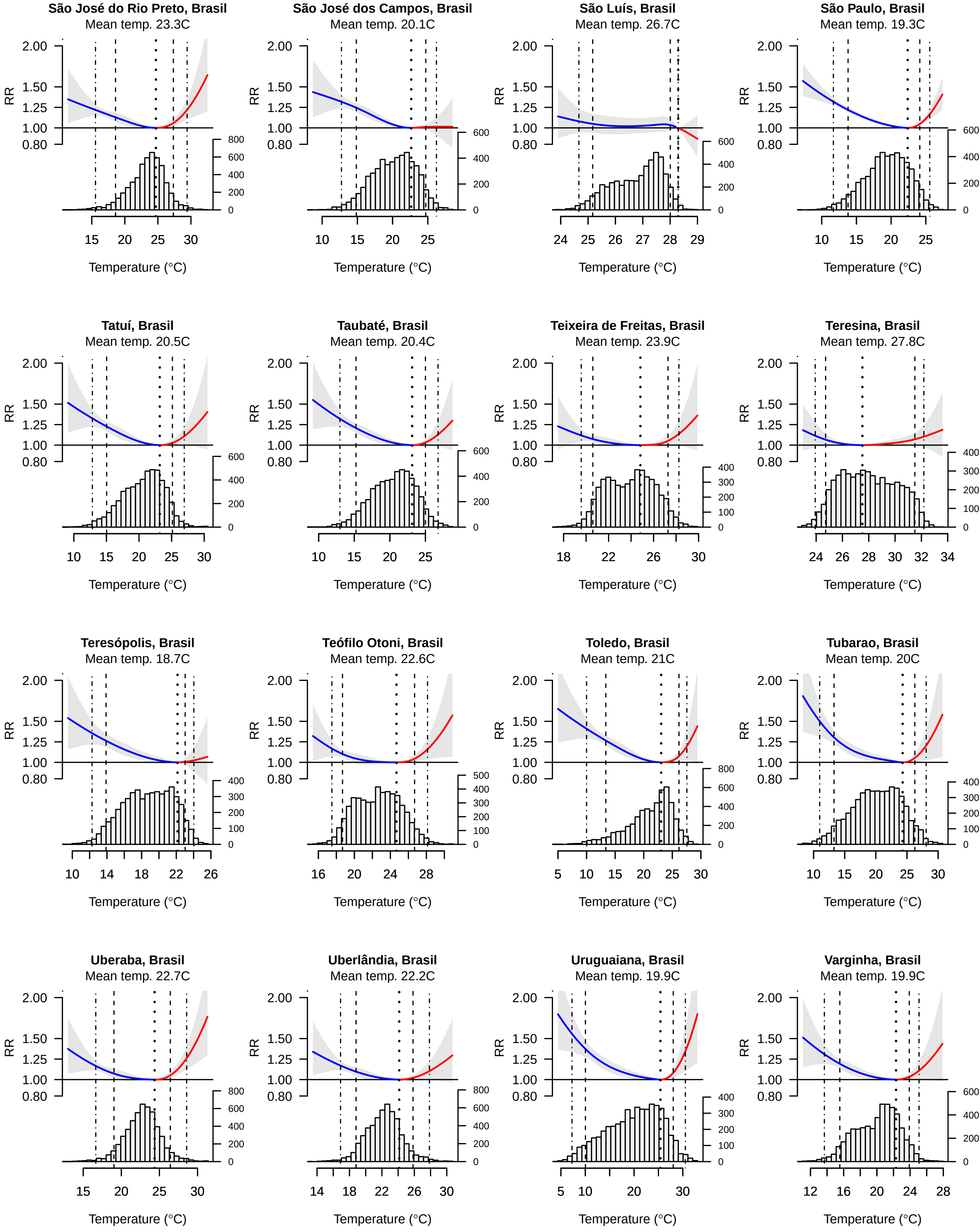

São José do Rio Preto, Brasil
Mean temp. 23.3C
São José dos Campos, Brasil
Mean temp. 20.1C
São Luís, Brasil
Mean temp. 26.7C
São Paulo, Brasil
Mean temp. 19.3C
Tatuí, Brasil
Mean temp. 20.5C
Taubaté, Brasil
Mean temp. 20.4C
Teixeira de Freitas, Brasil
Mean temp. 23.9C
Teresina, Brasil
Mean temp. 27.8C
Teresópolis, Brasil
Mean temp. 18.7C
Teófilo Otoni, Brasil
Mean temp. 22.6C
Toledo, Brasil
Mean temp. 21C
Tubarao, Brasil
Mean temp. 20C
Uberaba, Brasil
Mean temp. 22.7C
Uberlândia, Brasil
Mean temp. 22.2C
Uruguaiana, Brasil
Mean temp. 19.9C
Varginha, Brasil
Mean temp. 19.9C
RR
2.00
1.50
1.25
1.00
0.80
Temperature (°C)

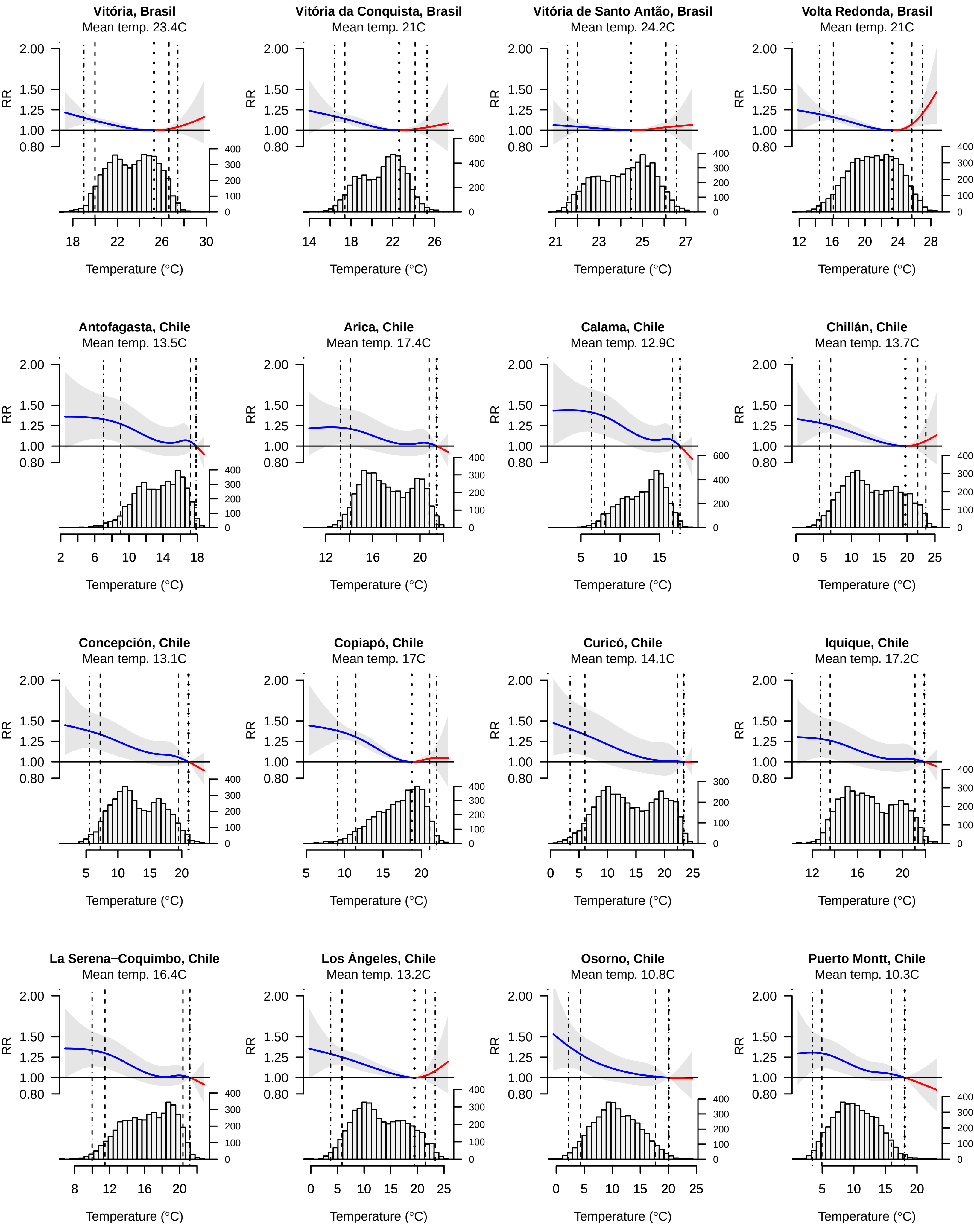
Vitória, Brasil
Mean temp. 23.4C
Vitória da Conquista, Brasil
Mean temp. 21C
Vitória de Santo Antão, Brasil
Mean temp. 24.2C
Volta Redonda, Brasil
Mean temp. 21C
Antofagasta, Chile
Mean temp. 13.5C
Arica, Chile
Mean temp. 17.4C
Calama, Chile
Mean temp. 12.9C
Chillán, Chile
Mean temp. 13.7C
Concepción, Chile
Mean temp. 13.1C
Copiapó, Chile
Mean temp. 17C
Curicó, Chile
Mean temp. 14.1C
Iquique, Chile
Mean temp. 17.2C
La Serena−Coquimbo, Chile
Mean temp. 16.4C
Los Ángeles, Chile
Mean temp. 13.2C
Osorno, Chile
Mean temp. 10.8C
Puerto Montt, Chile
Mean temp. 10.3C
RR
2.00
1.50
1.25
1.00
0.80
Temperature (°C)

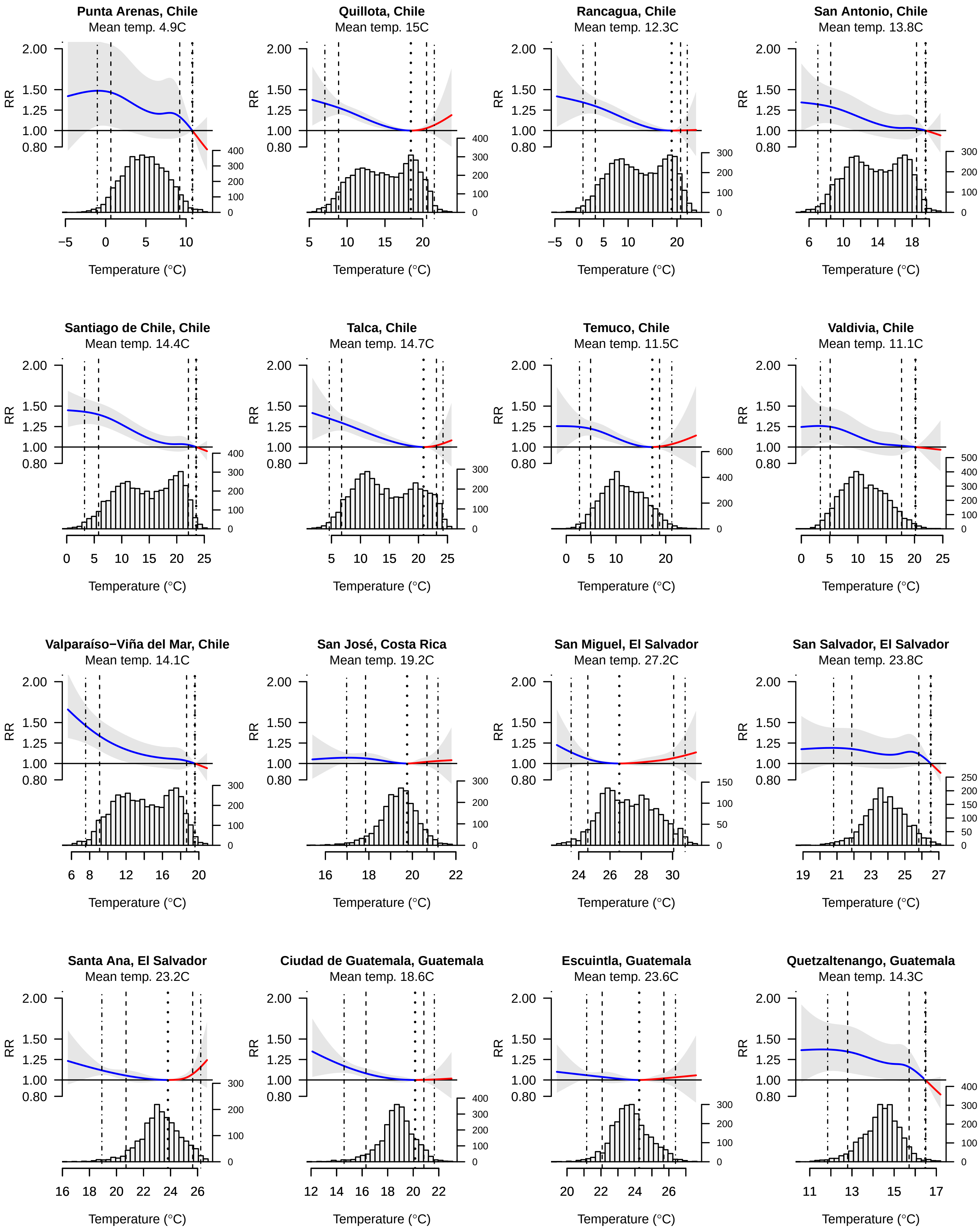
Punta Arenas, Chile
Mean temp. 4.9C
Quillota, Chile
Mean temp. 15C
Rancagua, Chile
Mean temp. 12.3C
San Antonio, Chile
Mean temp. 13.8C
Santiago de Chile, Chile
Mean temp. 14.4C
Talca, Chile
Mean temp. 14.7C
Temuco, Chile
Mean temp. 11.5C
Valdivia, Chile
Mean temp. 11.1C
Valparaíso−Viña del Mar, Chile
Mean temp. 14.1C
San José, Costa Rica
Mean temp. 19.2C
San Miguel, El Salvador
Mean temp. 27.2C
San Salvador, El Salvador
Mean temp. 23.8C
Santa Ana, El Salvador
Mean temp. 23.2C
Ciudad de Guatemala, Guatemala
Mean temp. 18.6C
Escuintla, Guatemala
Mean temp. 23.6C
Quetzaltenango, Guatemala
Mean temp. 14.3C
RR
Temperature (°C)

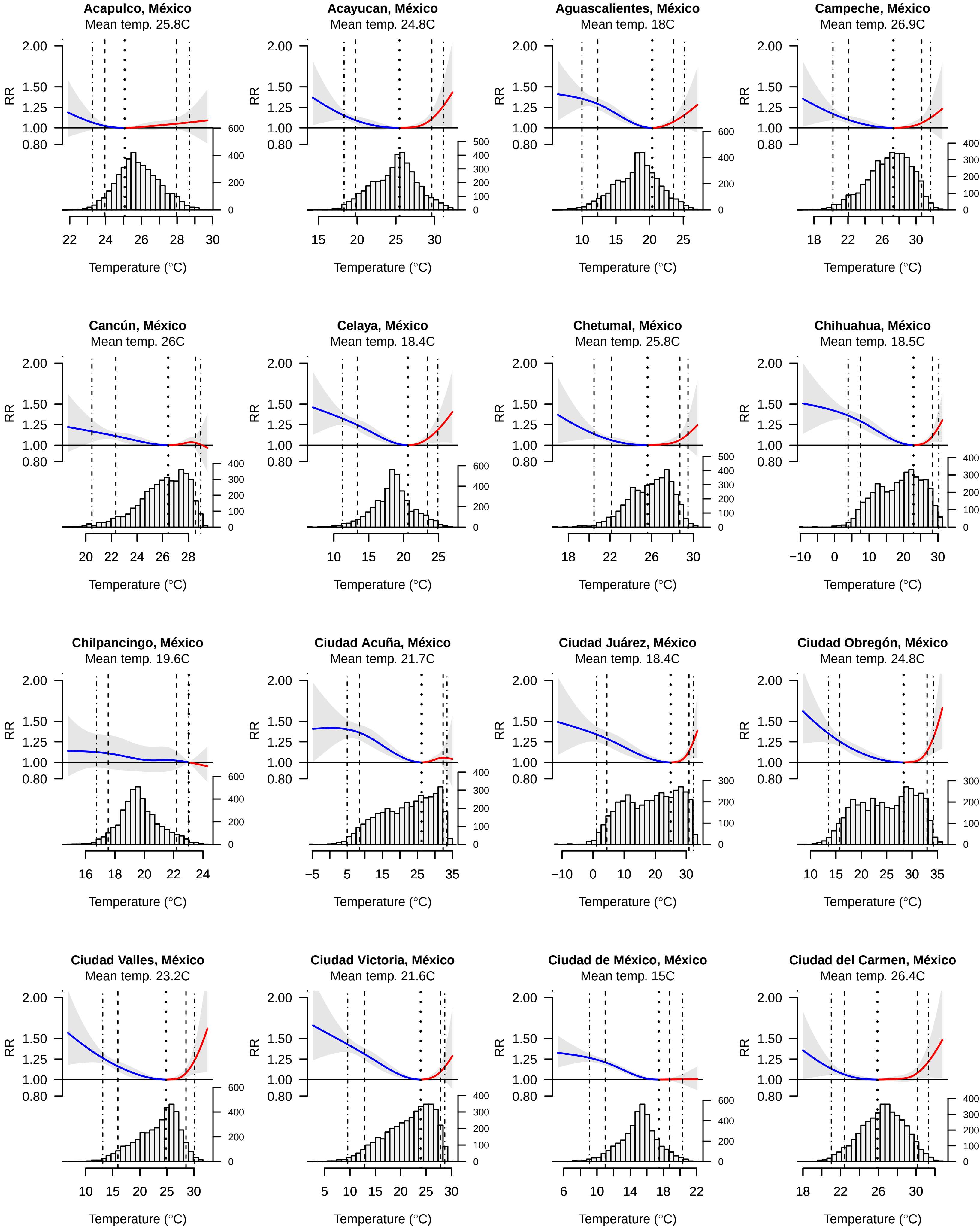

Acapulco, México
Mean temp. 25.8C
Acayucan, México
Mean temp. 24.8C
Aguascalientes, México
Mean temp. 18C
Campeche, México
Mean temp. 26.9C
Cancún, México
Mean temp. 26C
Celaya, México
Mean temp. 18.4C
Chetumal, México
Mean temp. 25.8C
Chihuahua, México
Mean temp. 18.5C
Chilpancingo, México
Mean temp. 19.6C
Ciudad Acuña, México
Mean temp. 21.7C
Ciudad Juárez, México
Mean temp. 18.4C
Ciudad Obregón, México
Mean temp. 24.8C
Ciudad Valles, México
Mean temp. 23.2C
Ciudad Victoria, México
Mean temp. 21.6C
Ciudad de México, México
Mean temp. 15C
Ciudad del Carmen, México
Mean temp. 26.4C
RR
Temperature (°C)

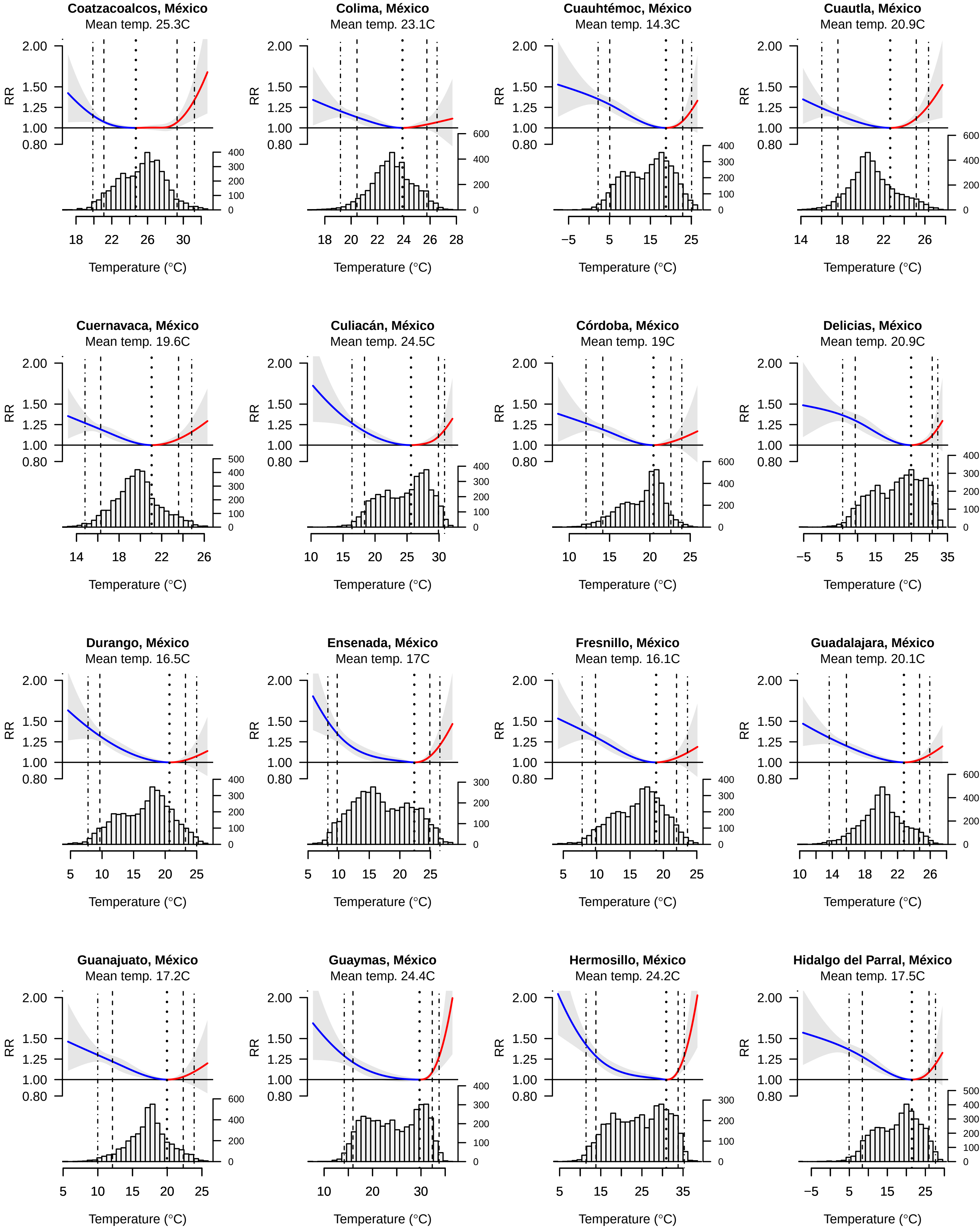
Coatzacoalcos, México
Mean temp. 25.3C
Colima, México
Mean temp. 23.1C
Cuauhtémoc, México
Mean temp. 14.3C
Cuautla, México
Mean temp. 20.9C
Cuernavaca, México
Mean temp. 19.6C
Culiacán, México
Mean temp. 24.5C
Córdoba, México
Mean temp. 19C
Delicias, México
Mean temp. 20.9C
Durango, México
Mean temp. 16.5C
Ensenada, México
Mean temp. 17C
Fresnillo, México
Mean temp. 16.1C
Guadalajara, México
Mean temp. 20.1C
Guanajuato, México
Mean temp. 17.2C
Guaymas, México
Mean temp. 24.4C
Hermosillo, México
Mean temp. 24.2C
Hidalgo del Parral, México
Mean temp. 17.5C
RR
Temperature (°C)

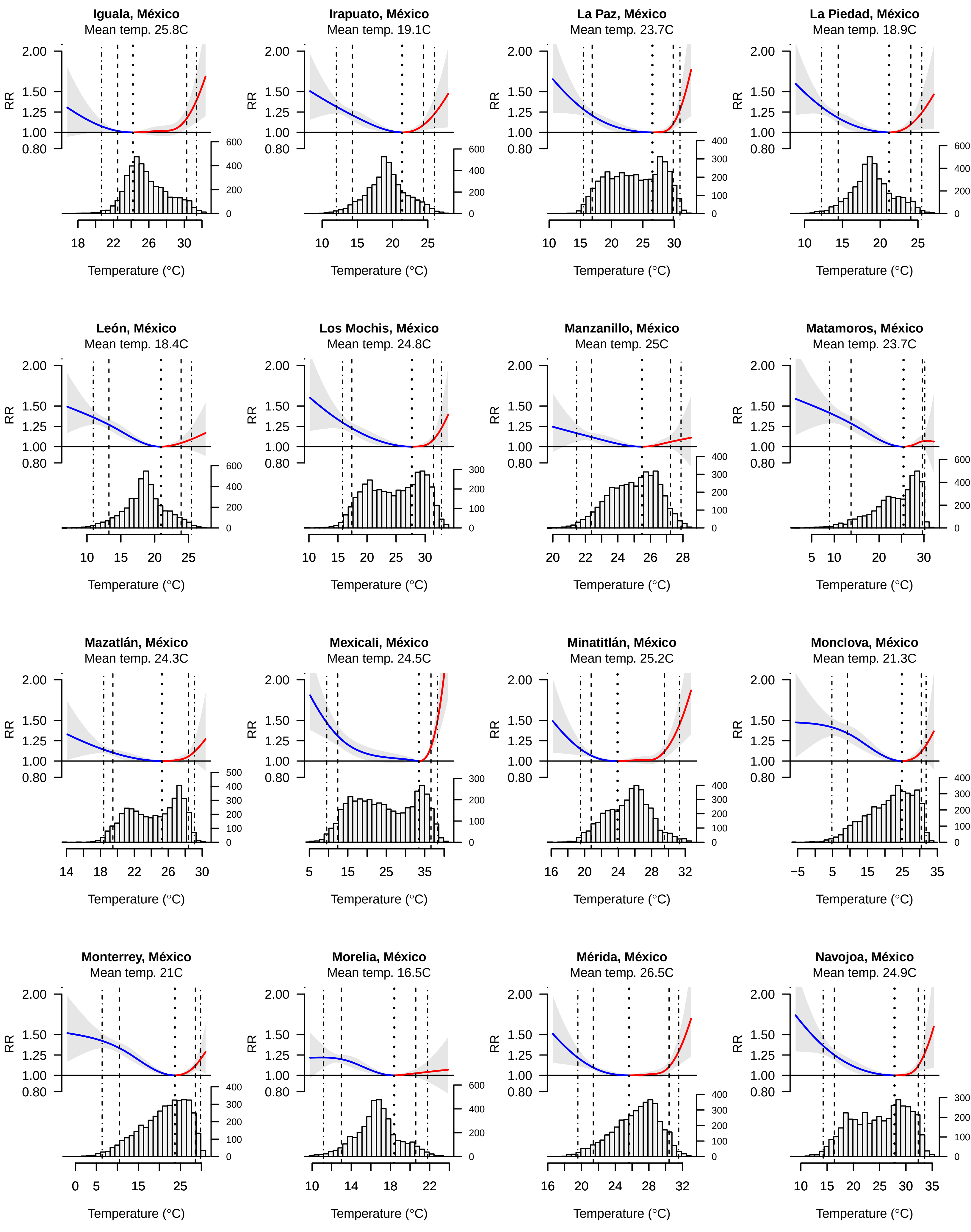
Iguala, México
Mean temp. 25.8C
Irapuato, México
Mean temp. 19.1C
La Paz, México
Mean temp. 23.7C
La Piedad, México
Mean temp. 18.9C
León, México
Mean temp. 18.4C
Los Mochis, México
Mean temp. 24.8C
Manzanillo, México
Mean temp. 25C
Matamoros, México
Mean temp. 23.7C
Mazatlán, México
Mean temp. 24.3C
Mexicali, México
Mean temp. 24.5C
Minatitlán, México
Mean temp. 25.2C
Monclova, México
Mean temp. 21.3C
Monterrey, México
Mean temp. 21C
Morelia, México
Mean temp. 16.5C
Mérida, México
Mean temp. 26.5C
Navojoa, México
Mean temp. 24.9C
RR
2.00
1.50
1.25
1.00
0.80
Temperature (°C)

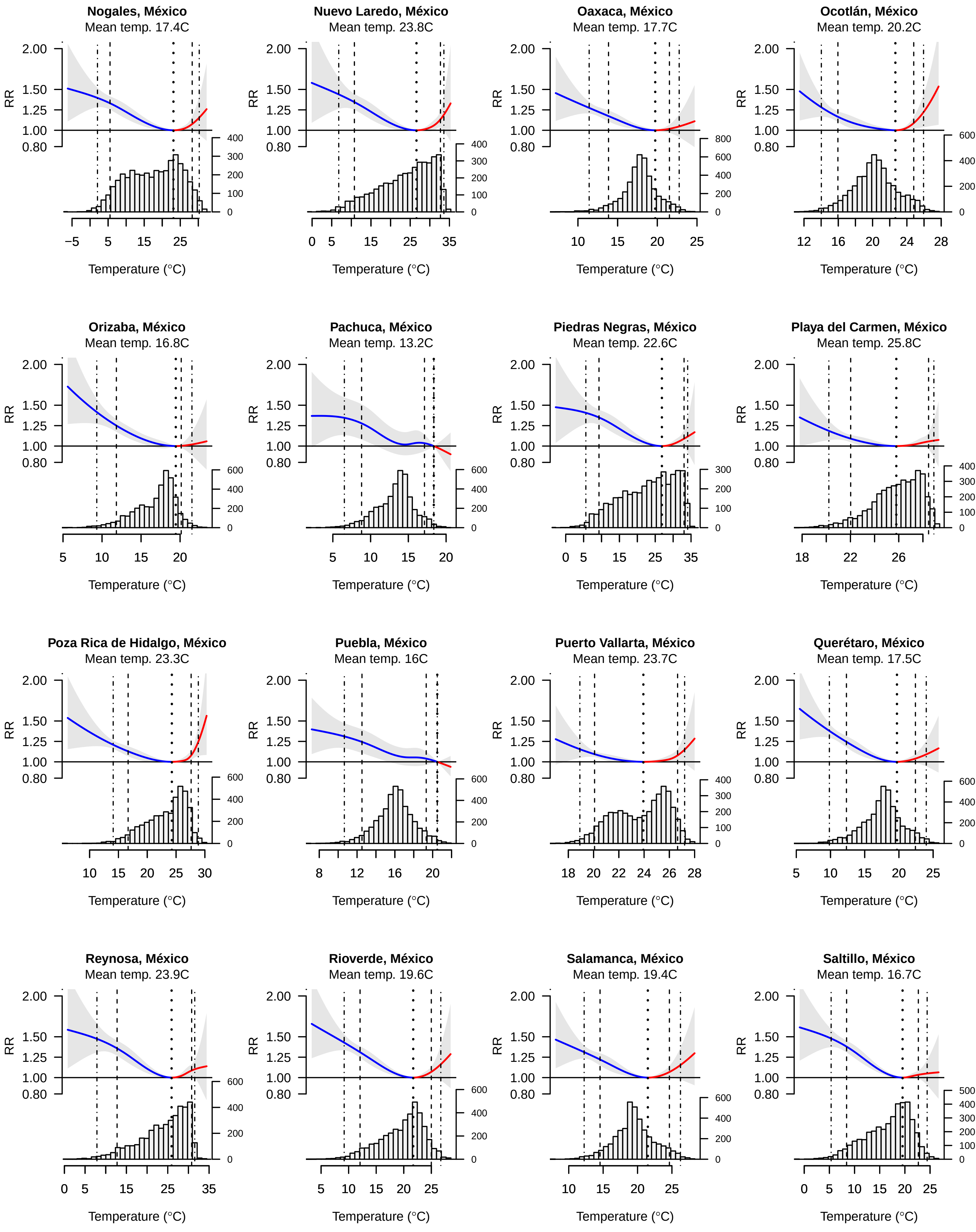
Nogales, México
Mean temp. 17.4C
Nuevo Laredo, México
Mean temp. 23.8C
Oaxaca, México
Mean temp. 17.7C
Ocotlán, México
Mean temp. 20.2C
Orizaba, México
Mean temp. 16.8C
Pachuca, México
Mean temp. 13.2C
Piedras Negras, México
Mean temp. 22.6C
Playa del Carmen, México
Mean temp. 25.8C
Poza Rica de Hidalgo, México
Mean temp. 23.3C
Puebla, México
Mean temp. 16C
Puerto Vallarta, México
Mean temp. 23.7C
Querétaro, México
Mean temp. 17.5C
Reynosa, México
Mean temp. 23.9C
Rioverde, México
Mean temp. 19.6C
Salamanca, México
Mean temp. 19.4C
Saltillo, México
Mean temp. 16.7C
RR
Temperature (°C)

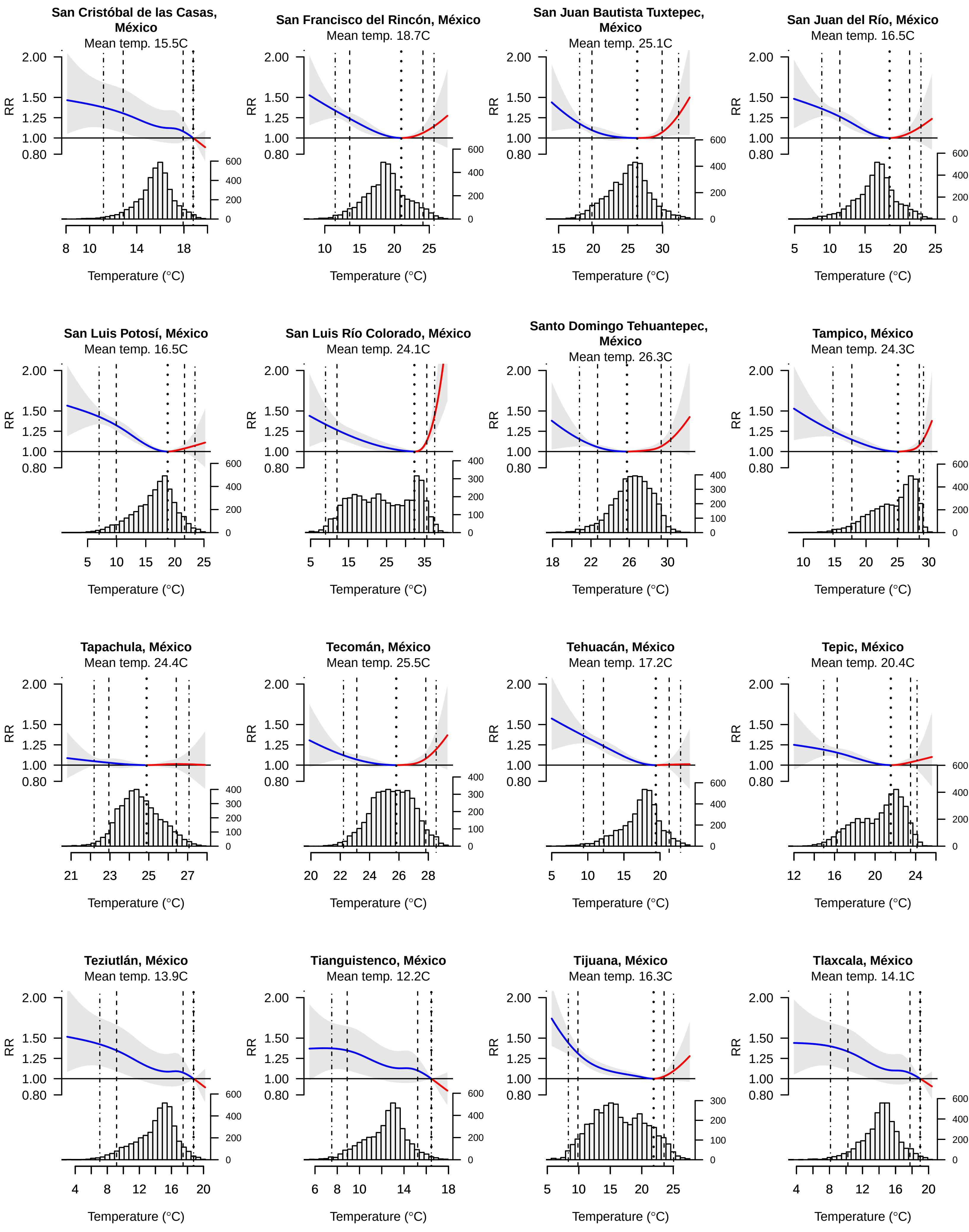
San Cristóbal de las Casas, México
Mean temp. 15.5C
San Francisco del Rincón, México
Mean temp. 18.7C
San Juan Bautista Tuxtepec, México
Mean temp. 25.1C
San Juan del Río, México
Mean temp. 16.5C
San Luis Potosí, México
Mean temp. 16.5C
San Luis Río Colorado, México
Mean temp. 24.1C
Santo Domingo Tehuantepec, México
Mean temp. 26.3C
Tampico, México
Mean temp. 24.3C
Tapachula, México
Mean temp. 24.4C
Tecomán, México
Mean temp. 25.5C
Tehuacán, México
Mean temp. 17.2C
Tepic, México
Mean temp. 20.4C
Teziutlán, México
Mean temp. 13.9C
Tianguistenco, México
Mean temp. 12.2C
Tijuana, México
Mean temp. 16.3C
Tlaxcala, México
Mean temp. 14.1C
RR
Temperature (°C)

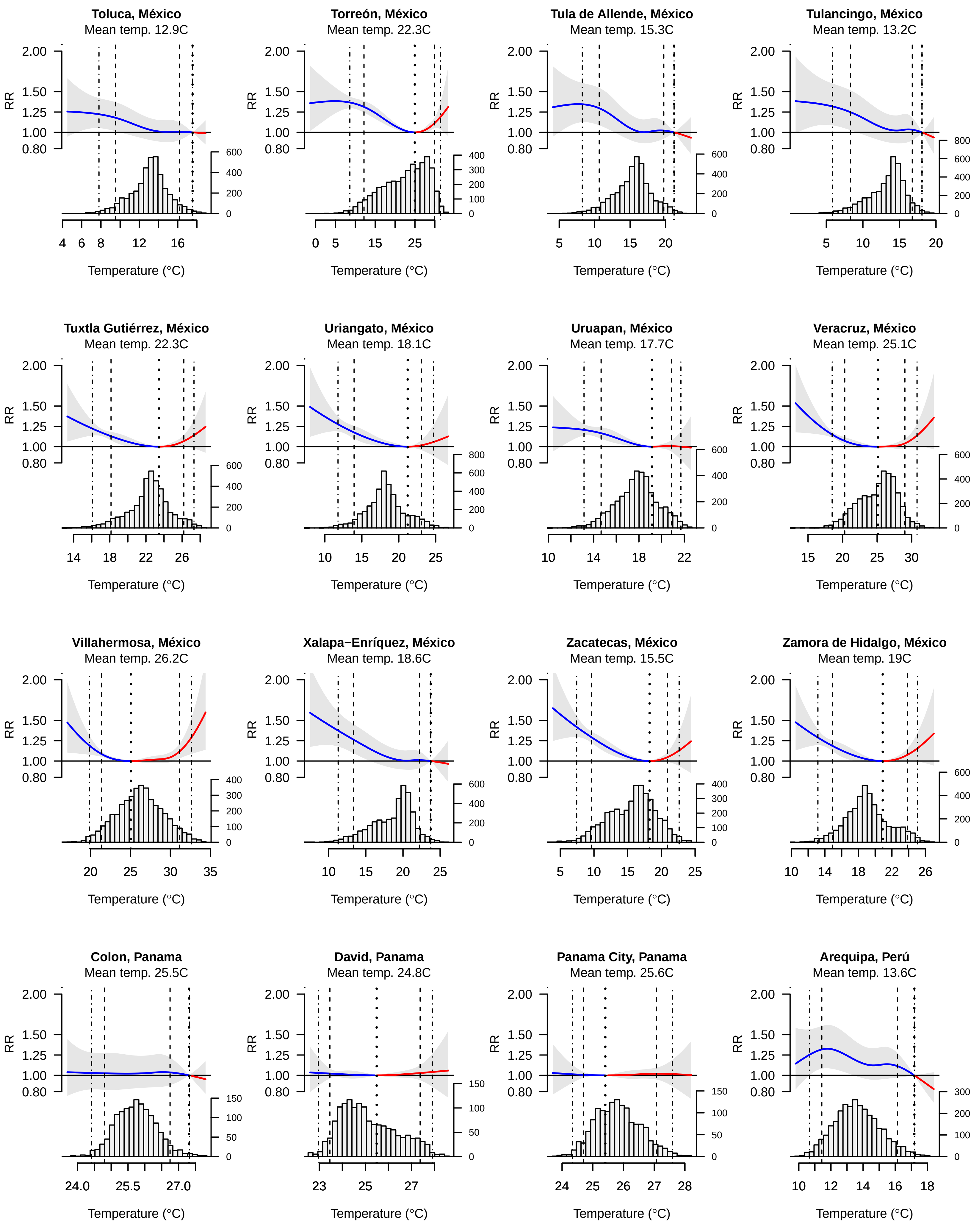
Toluca, México
Mean temp. 12.9C
Torreón, México
Mean temp. 22.3C
Tula de Allende, México
Mean temp. 15.3C
Tulancingo, México
Mean temp. 13.2C
Tuxtla Gutiérrez, México
Mean temp. 22.3C
Uriangato, México
Mean temp. 18.1C
Uruapan, México
Mean temp. 17.7C
Veracruz, México
Mean temp. 25.1C
Villahermosa, México
Mean temp. 26.2C
Xalapa−Enríquez, México
Mean temp. 18.6C
Zacatecas, México
Mean temp. 15.5C
Zamora de Hidalgo, México
Mean temp. 19C
Colon, Panama
Mean temp. 25.5C
David, Panama
Mean temp. 24.8C
Panama City, Panama
Mean temp. 25.6C
Arequipa, Perú
Mean temp. 13.6C
RR
2.00
1.50
1.25
1.00
0.80
Temperature (°C)

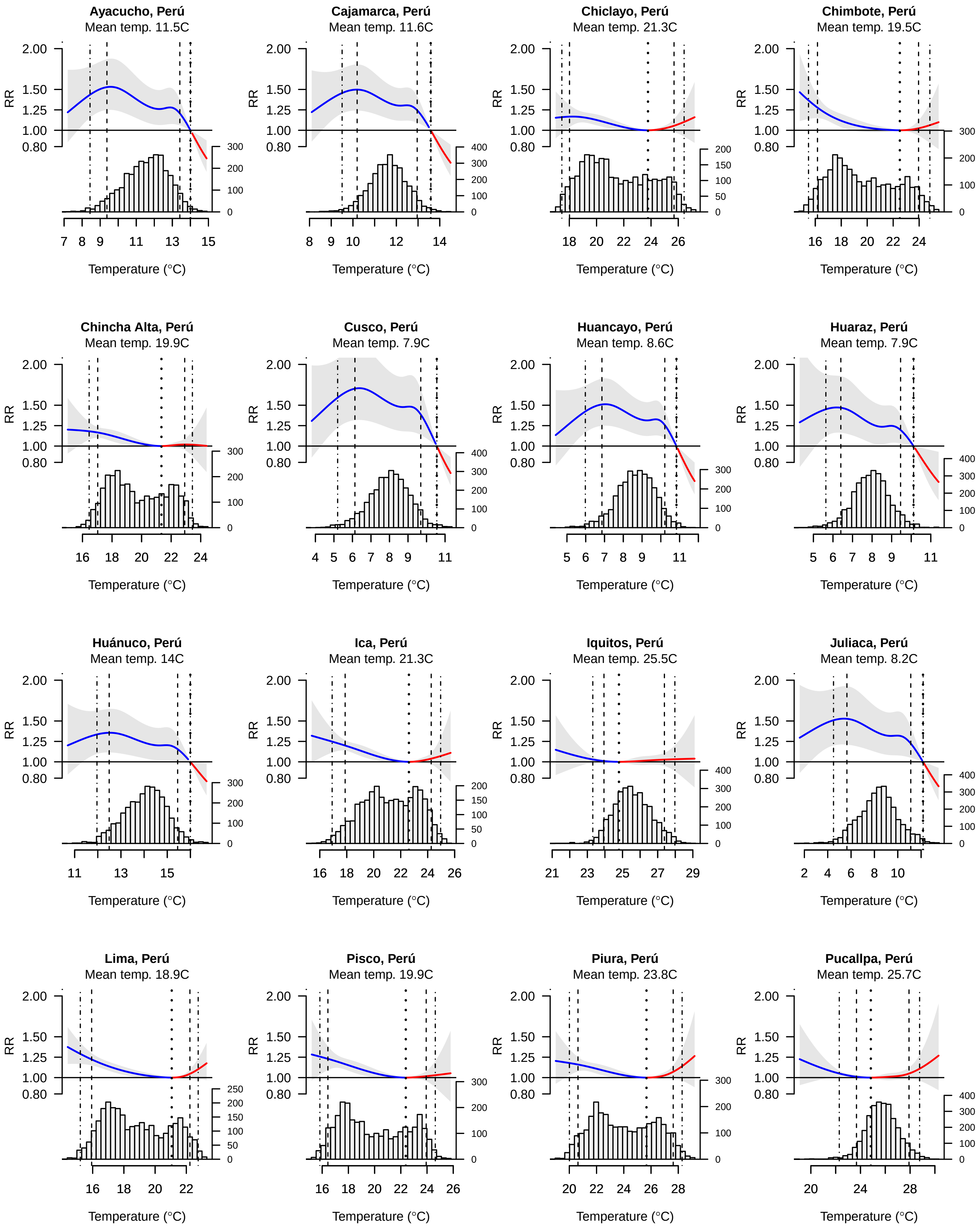
Ayacucho, Perú
Mean temp. 11.5C
Cajamarca, Perú
Mean temp. 11.6C
Chiclayo, Perú
Mean temp. 21.3C
Chimbote, Perú
Mean temp. 19.5C
Chincha Alta, Perú
Mean temp. 19.9C
Cusco, Perú
Mean temp. 7.9C
Huancayo, Perú
Mean temp. 8.6C
Huaraz, Perú
Mean temp. 7.9C
Huánuco, Perú
Mean temp. 14C
Ica, Perú
Mean temp. 21.3C
Iquitos, Perú
Mean temp. 25.5C
Juliaca, Perú
Mean temp. 8.2C
Lima, Perú
Mean temp. 18.9C
Pisco, Perú
Mean temp. 19.9C
Piura, Perú
Mean temp. 23.8C
Pucallpa, Perú
Mean temp. 25.7C
RR
Temperature (°C)

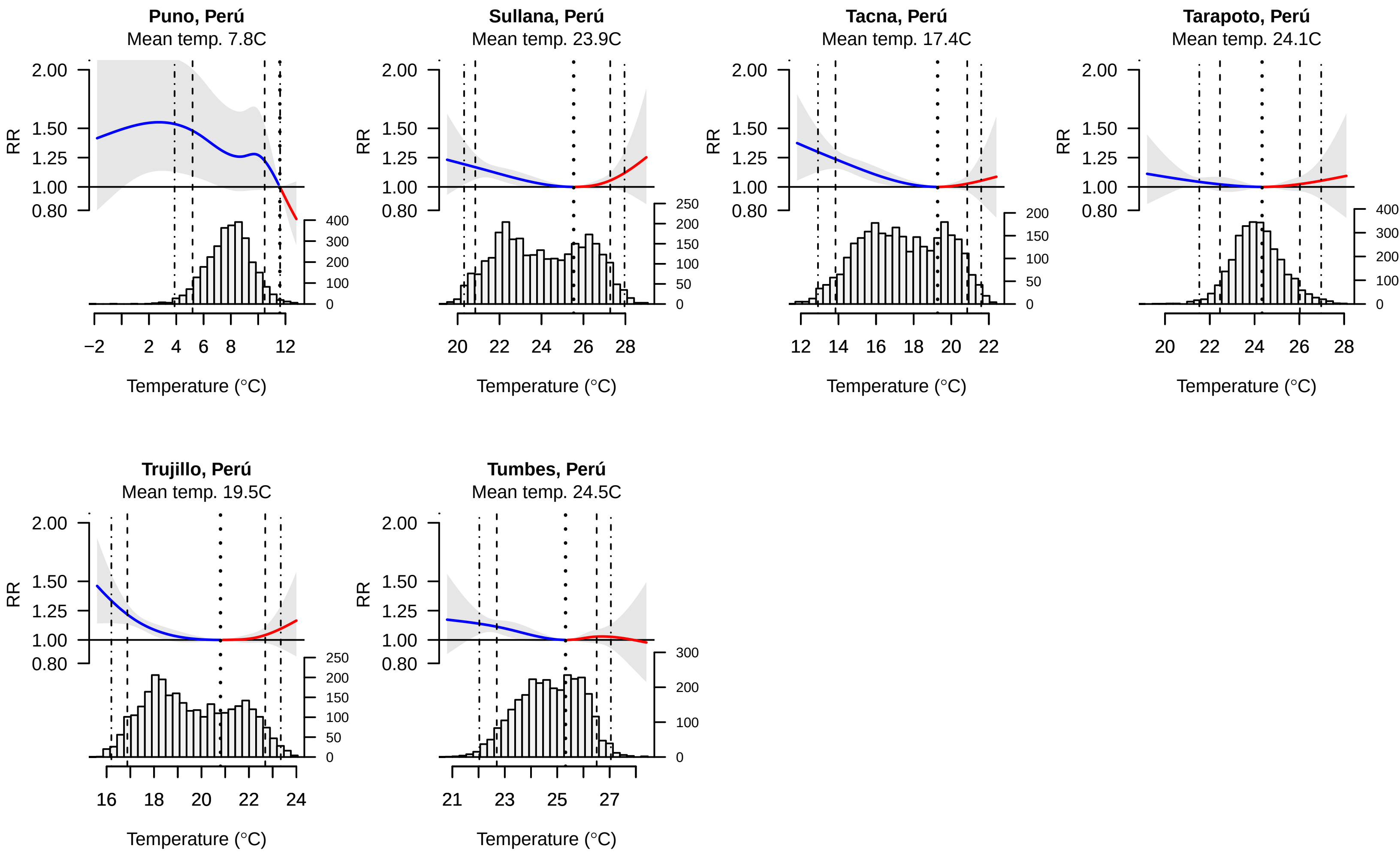

Puno, Perú
Mean temp. 7.8C
Sullana, Perú
Mean temp. 23.9C
Tacna, Perú
Mean temp. 17.4C
Tarapoto, Perú
Mean temp. 24.1C
Trujillo, Perú
Mean temp. 19.5C
Tumbes, Perú
Mean temp. 24.5C
RR
2.00
1.50
1.25
1.00
0.80
Temperature (°C)

Supplementary Table 1. City-specific daily temperature distributions and temperature-mortality associations

			Temperature			Temperature Association with Mortality		
Country	City	Total Number of Deaths	5th Percentile Temperature	Median Temperature	95th Percentile Temperature	Minimum Mortality Temperature ^{0}C	Excess Death Fraction All Cold (%)	Excess Death Fraction All Heat (%)
**Argentina**	Buenos Aires	172311	7.9	17.0	25.9	23.5	15.2	2
	Comodoro Rivadavia	7682	3.6	11.8	21.0	18	5.8	-1
	Concordia	8562	8.9	19.2	27.5	24.8	8.2	1.2
	Corrientes	16278	11.7	22.1	29.4	26.2	17.7	1.2
	Córdoba	84196	7.9	17.7	25.7	22.2	6.6	1.5
	Formosa	10354	12.4	23.4	30.5	26.7	16.4	-1.6
	La Rioja	7363	8.7	19.4	28.7	24.6	11.2	2.4
	Mendoza	57175	5.3	15.9	26.0	22.3	11.5	1.6
	Neuquén-Plottier-Cipolletti	28411	5.3	15.3	27.0	23.2	9.5	0.7
	Paraná	18864	8.8	19.0	27.3	24.7	8.1	1.5
	Posadas	14997	12.0	21.9	28.2	25.4	9.6	2
	Rawson - Trelew	5745	5.2	14.2	24.4	21	8.5	0.6
	Resistencia	18941	11.6	22.2	29.8	26.6	14.6	-0.3
	Rosario	90402	8.5	18.5	26.9	24.5	10.7	1.4
	Río Cuarto	16680	7.9	17.6	26.5	22.9	3	2.1
	Río Gallegos	3092	-0.4	6.8	14.4	16.7	-23.8	0.1
	Salta	24357	7.9	16.5	22.1	19	5.2	0.7
	San Carlos de Bariloche	5002	-1.2	5.2	16.8	18.8	-4.8	0.3
	San Fernando del Valle de Catamarca	8270	8.6	19.6	28.1	24.3	7.5	-0.8
	San Juan	24962	7.3	17.9	26.9	22.6	6.3	1.5
	San Luis	8448	7.3	17.8	27.7	24.4	29.7	1.4
	San Miguel de Tucumán-Tafí Viejo	47772	10.0	19.1	25.9	23.8	8.9	1.8

			Temperature			Temperature Association with Mortality		
Country	City	Total Number of Deaths	5th Percentile Temperature	Median Temperature	95th Percentile Temperature	Minimum Mortality Temperature ^{0}C	Excess Death Fraction All Cold (%)	Excess Death Fraction All Heat (%)
	San Rafael	10313	5.5	16.4	27.3	23.2	-2.9	1.5
	San Salvador de Jujuy	13011	8.0	15.6	20.6	17.7	2.5	0.6
	Santa Fe	31557	9.4	19.5	27.8	25.1	6.7	1.5
	Santa Rosa-Toay	5910	6.5	16.5	27.0	23.8	15.5	2
	Santiago del Estero- La Banda	19492	12.4	22.7	31.1	27.3	3.2	1.2
	Villa Mercedes	6426	7.4	17.4	26.9	23.9	-9.7	0.6
**Brasil**	Alagoinhas	12167	21.5	24.7	27.5	25.2	1.8	-1.2
	Angra dos Reis	11764	16.8	21.6	26.2	24.6	-1.1	-0.7
	Anápolis	28352	19.7	22.0	25.8	23.8	2.2	-0.2
	Apucarana	10514	14.1	21.0	24.8	22.5	3	2.3
	Aracaju	58215	23.2	25.5	27.3	25.9	3.6	0.6
	Araguari	10117	18.8	22.4	26.0	23.9	-10.5	0.9
	Araguaína	9928	24.2	26.3	29.1	25.8	3	6
	Arapiraca	17849	21.6	24.9	27.5	25.7	5.1	-2.2
	Arapongas	14252	14.3	21.2	25.0	23.1	3.4	0.5
	Araraquara	22801	17.3	22.5	26.2	23.8	-0.3	0.6
	Araras	10922	16.1	21.7	25.6	23.7	0.4	2
	Araruama	21423	19.4	23.0	26.7	24.9	11.1	1.7
	Araçatuba	18892	18.7	24.4	28.2	25	1.2	3.7
	Atibaia	13875	14.0	19.6	23.7	21.9	5.6	2.4
	Balneário Camboriú	10988	14.4	20.5	25.8	23.9	-2.3	-1.1
	Barbacena	12760	13.7	18.7	22.2	20.9	11.4	0.2
	Barreiras	7798	22.3	24.8	28.9	24.2	1.2	4.8
	Barretos	13154	19.1	23.8	27.6	25.2	7.1	1.8

			Temperature			Temperature Association with Mortality		
Country	City	Total Number of Deaths	5th Percentile Temperature	Median Temperature	95th Percentile Temperature	Minimum Mortality Temperature ^{0}C	Excess Death Fraction All Cold (%)	Excess Death Fraction All Heat (%)
	Bauru	32505	16.8	22.6	26.6	24	3.6	1.9
	Belo Horizonte	357006	16.4	20.9	24.3	22	2	0.6
	Belém	150465	24.9	26.6	28.3	28.7	8.4	0
	Bento Goncalves	12198	8.7	17.8	23.5	21.3	16.7	0.9
	Birigui	9026	18.6	24.3	28.1	25.4	6.3	1.9
	Blumenau	34585	13.1	19.7	25.2	23.1	0.5	0.4
	Boa Vista	14681	24.4	26.7	29.8	26.3	-0.3	2.1
	Botucatu	11829	14.7	20.7	24.6	23.3	2.9	0.4
	Bragança Paulista	14621	14.2	19.9	23.9	22.3	9.7	1.4
	Brasília	178255	19.0	21.6	25.4	23.4	2	0.6
	Brusque	8200	12.9	19.6	25.1	23.3	-2.5	-1.4
	Cabo Frio	24696	19.6	23.0	26.4	25.5	9.1	0.4
	Cachoeiro de Itapemirim	16980	18.1	22.5	27.1	25.3	4.8	1.3
	Campina Grande	40634	21.1	23.7	25.4	25.9	7.4	0
	Campinas	219551	15.8	21.4	25.5	24	6.3	0.6
	Campo Grande	59982	17.6	24.1	27.6	25.7	4	-0.5
	Campos dos Goytacazes	49472	19.7	23.8	27.8	25.6	4.4	0.7
	Caraguatatuba	13929	16.8	21.4	26.1	24.1	-5.6	2.6
	Caruaru	27763	20.2	23.0	25.0	23.8	5	0.5
	Cascavel	20823	12.3	20.9	24.9	22.9	4.8	0.4
	Castanhal	11036	24.5	26.2	28.5	25.8	6.2	6.6
	Catanduva	13262	18.3	23.5	27.2	24.5	2	3.2
	Caxias	10829	24.5	27.5	30.9	26.9	-0.1	0
	Caxias do Sul	32310	8.4	17.4	22.8	19.7	5.2	-0.3

			Temperature			Temperature Association with Mortality		
Country	City	Total Number of Deaths	5th Percentile Temperature	Median Temperature	95th Percentile Temperature	Minimum Mortality Temperature ^{0}C	Excess Death Fraction All Cold (%)	Excess Death Fraction All Heat (%)
	Chapecó	11170	10.6	19.8	24.4	22.2	7.4	-0.1
	Conselheiro Lafaiete	9597	14.6	19.5	22.9	21.5	-4.6	-0.6
	Criciúma	23285	12.7	19.9	25.6	23.7	2.3	1.2
	Cuiabá	60535	22.0	26.5	30.0	26.4	-0.5	0.6
	Curitiba	223491	10.8	17.5	22.5	19.9	6.3	0.3
	Divinópolis	16391	17.2	21.5	25.1	23.8	10.5	0.1
	Dourados	15753	15.9	24.3	28.3	24.8	2.9	0.1
	Feira de Santana	42793	21.1	24.6	27.8	25.3	2.4	1.1
	Florianópolis	55776	14.2	20.4	25.8	23.8	7.5	0.4
	Fortaleza	246596	25.2	26.7	27.7	28.1	8.1	0
	Foz do Iguaçu	20321	13.6	23.1	28.0	25.5	2.1	-1.1
	Franca	26382	17.1	21.6	25.0	23.5	4.5	0.1
	Garanhuns	11735	18.9	21.9	24.3	23	9.1	-1.3
	Goiânia	148759	20.8	23.2	27.3	24.6	2	1.2
	Governador Valadares	23969	20.3	24.2	28.1	23.9	1	1.7
	Guarapari	8318	19.7	23.3	26.7	24.5	-8.4	-2.5
	Guarapuava	14754	10.3	17.9	21.8	19.7	-1	-0.4
	Guaratinguetá	25657	15.4	20.8	25.1	23.2	2.4	-0.2
	Ilhéus	16926	21.2	24.0	26.2	24.4	4	2.8
	Imperatriz	21849	24.5	26.8	29.8	27.4	1.2	-2.7
	Ipatinga	38200	18.8	22.8	26.7	24.4	6.8	1.3
	Itabira	8137	16.1	20.5	23.9	22.3	6.2	-0.5
	Itabuna	21001	20.7	23.6	25.8	25.1	10.8	0.4
	Itajaí	23982	14.8	20.8	26.0	24.3	-1.2	0.4

			Temperature			Temperature Association with Mortality		
**Country**	**City**	**Total Number of Deaths**	**5th Percentile Temperature**	**Median Temperature**	**95th Percentile Temperature**	**Minimum Mortality Temperature ^{0}C**	**Excess Death Fraction All Cold (%)**	**Excess Death Fraction All Heat (%)**
	Itapetininga	13360	14.0	20.1	24.4	22.7	9.9	0.2
	Jaraguá do Sul	11859	13.6	20.1	25.6	23.6	13.1	0.4
	Jaú	13276	16.7	22.4	26.5	23.7	2.1	3.8
	Jequié	13364	19.4	23.0	25.7	23.5	-3.1	1
	Ji-Paraná	8421	23.8	25.7	28.8	26.2	5.2	-0.6
	Joinville	35248	14.4	20.8	26.2	24.2	1.6	1.2
	João Pessoa	83786	23.9	25.8	27.3	25.5	0.2	1.3
	Juazeiro do Norte	34505	23.1	25.6	29.2	26.8	1.6	0.4
	Juiz de Fora	52045	15.3	20.3	24.3	22.9	4.3	-0.1
	Jundiaí	48893	14.2	19.9	23.9	21.9	4.4	1.2
	Lages (Lajes)	14922	8.3	16.4	21.6	19.1	0.8	0.6
	Limeira	25482	16.2	22.0	26.0	24.4	9	0.7
	Linhares	10155	20.6	24.0	27.0	25.1	6.9	1.8
	Londrina	55611	14.9	21.7	25.6	23	4.3	1.6
	Macapá	25510	24.4	26.3	28.5	25.8	0.9	2.4
	Macaé	14119	19.0	23.0	27.1	24.1	-3.1	0.4
	Maceió	87910	23.2	25.3	27.0	25.7	-0.2	0
	Manaus	114662	24.8	26.2	28.8	25.4	-0.1	-0.6
	Marabá	15787	24.2	26.3	29.0	27.1	12.2	-2
	Maringá	38724	15.1	22.6	26.6	23.9	1.9	0.8
	Marília	19942	17.0	22.9	26.8	24.2	5.4	1.5
	Mogi Guaçu (Moji Guaçu)	21683	16.3	21.8	25.7	23.6	7.2	0.9
	Montes Claros	25378	19.4	23.0	27.1	25.1	1.9	1.7
	Mossoró	18513	25.7	27.9	29.3	27.3	-0.5	1.8

			Temperature			Temperature Association with Mortality		
**Country**	**City**	**Total Number of Deaths**	**5th Percentile Temperature**	**Median Temperature**	**95th Percentile Temperature**	**Minimum Mortality Temperature ^{0}C**	**Excess Death Fraction All Cold (%)**	**Excess Death Fraction All Heat (%)**
	Natal	85164	24.2	26.0	27.3	25	0.8	3.5
	Nova Friburgo	20192	13.6	18.5	22.6	21.6	19	0.8
	Ourinhos	10196	16.4	22.7	26.9	24	-0.3	2.1
	Palmas	9187	24.3	26.6	30.2	26	0.2	3
	Paranaguá	12513	15.7	21.2	26.4	24.3	4.3	1.1
	Parauapebas	7419	23.6	25.7	28.5	25.7	2.6	1.7
	Parnaíba	11441	25.7	27.8	28.9	26.9	0.5	2.5
	Parobe	13586	10.2	19.1	25.0	22.7	13.3	0.6
	Passo Fundo	17382	9.2	18.7	23.8	20.7	3.1	-0.4
	Passos	10035	16.8	21.7	25.2	22.9	4.3	2.4
	Patos de Minas	10992	18.2	21.7	25.3	23.2	7.7	1.8
	Pelotas	41495	10.1	18.8	25.1	23.2	5.6	-1.2
	Petrolina	33104	23.3	26.4	29.8	27.2	0.9	1.4
	Petrópolis	35441	14.6	19.6	23.9	22.5	5.9	-0.4
	Piracicaba	33655	16.1	22.1	26.1	24	1.9	1.5
	Ponta Grossa	28686	11.7	18.7	23.0	21.5	9.6	0
	Porto Alegre	350444	11.1	19.8	26.0	23.8	5.5	1.3
	Porto Seguro	7656	21.5	24.3	26.6	24.7	3.1	0.5
	Porto Velho	30179	24.3	25.9	28.5	24.8	-0.3	4.9
	Pouso Alegre	9869	15.0	20.0	23.7	22.4	8.7	1.5
	Poços de Caldas	14490	13.6	18.6	21.8	20.6	11.3	1.3
	Presidente Prudente	21703	17.1	23.8	27.7	24.5	3	2.9
	Recife	312731	23.5	25.6	27.3	26	-0.3	0.2
	Resende	14138	15.5	20.8	25.4	23.3	9.6	0.1

			Temperature			Temperature Association with Mortality		
**Country**	**City**	**Total Number of Deaths**	**5th Percentile Temperature**	**Median Temperature**	**95th Percentile Temperature**	**Minimum Mortality Temperature ^{0}C**	**Excess Death Fraction All Cold (%)**	**Excess Death Fraction All Heat (%)**
	Ribeirão Preto	51462	17.9	23.0	26.7	24	1.9	2
	Rio Branco	22061	23.0	25.4	28.0	25.8	2.8	0.9
	Rio Claro	20396	16.2	21.8	25.8	24.4	12.9	-0.2
	Rio Grande	23032	10.9	18.8	24.8	23.1	-2.3	0.6
	Rio Verde	11892	20.0	23.3	26.8	25	-4.2	1.4
	Rio das Ostras	9353	18.8	23.0	27.2	25.3	4.7	-0.2
	Rio de Janeiro	1282138	18.4	23.0	28.1	23.3	2.1	1.2
	Rondonópolis	14730	22.5	26.0	29.4	26.6	-0.2	-2.2
	Salvador	239702	23.0	25.3	27.3	28	5.5	0
	Santa Cruz do Sul	11848	10.1	19.4	25.8	23.3	9.7	1.1
	Santa Maria	25688	9.6	19.6	26.3	23.7	9.6	-0.7
	Santarém	16817	24.9	26.7	29.5	26.9	-1.5	-0.8
	Santos	168093	16.8	21.7	26.7	23.6	1.5	1.3
	Sertãozinho	8653	18.4	23.4	27.3	25	6.5	2
	Sete Lagoas	16699	17.5	21.7	25.4	23.8	2.7	0.7
	Sobral	13055	24.9	27.4	29.2	27	2.7	-1.6
	Sorocaba	62281	14.8	20.7	24.9	23.8	6.8	0.7
	São Carlos	20224	16.1	21.3	25.0	23.4	7.2	0.3
	São José do Rio Preto	45225	18.6	23.7	27.3	24.7	5.5	2.5
	São José dos Campos	69062	14.9	20.4	24.7	22.6	7.8	0.3
	São Luís	83401	25.2	27.0	28.0	28.3	7.7	-0.1
	São Paulo	1594830	13.8	19.6	24.1	22.4	5.6	0.6
	Tatuí	10439	15.0	21.0	25.1	23.2	5.9	2.3
	Taubaté	38986	15.2	20.7	25.0	23.1	4.4	0.7

			Temperature			Temperature Association with Mortality		
**Country**	**City**	**Total Number of Deaths**	**5th Percentile Temperature**	**Median Temperature**	**95th Percentile Temperature**	**Minimum Mortality Temperature ^{0}C**	**Excess Death Fraction All Cold (%)**	**Excess Death Fraction All Heat (%)**
	Teixeira de Freitas	10601	20.6	24.1	27.3	24.8	-7.1	-1.5
	Teresina	69439	24.7	27.9	31.5	27.5	1.5	1.5
	Teresópolis	17477	13.9	18.9	23.0	22.1	21.5	0
	Teófilo Otoni	13949	18.7	22.7	26.7	24.7	-4.1	0.5
	Toledo	8671	13.4	22.0	26.2	23.1	-1.5	3.6
	Tubarao	11146	13.3	20.3	26.3	24.3	17.7	0.4
	Uberaba	27999	19.0	22.9	26.4	24.4	0	1.3
	Uberlândia	43566	18.8	22.3	25.8	24.1	3.4	-0.1
	Uruguaiana	12981	10.0	20.6	28.0	25.4	6.9	1.3
	Varginha	10080	15.5	20.4	23.9	22.3	-3.7	1.4
	Vitória	123055	20.0	23.5	26.6	25.3	5.2	0
	Vitória da Conquista	25399	17.4	21.3	24.1	22.6	6.1	-0.9
	Vitória de Santo Antão	12508	22.0	24.3	26.1	24.5	-5.8	2.4
	Volta Redonda	55448	16.1	21.2	25.7	23.3	5.8	1.2
**Chile**	Antofagasta	21398	9.0	13.7	17.2	17.9	0.8	-0.1
	Arica	13061	14.1	17.2	20.8	21.4	10.8	-0.1
	Calama	7436	8.0	13.4	16.7	17.6	23.2	0
	Chillán	14574	6.3	13.1	21.9	19.7	8	-0.6
	Concepción	62576	7.2	12.6	19.5	21.1	28.5	-0.2
	Copiapó	8457	11.5	17.5	21.1	18.8	9.1	-1
	Curicó	9254	6.0	13.7	22.3	23.4	45.8	-0.1
	Iquique	14099	13.6	16.9	21.1	21.9	21.2	-0.1
	La Serena-Coquimbo	23622	11.5	16.6	20.4	21.2	24.3	-0.1
	Los Ángeles	12309	5.9	12.6	21.5	19.5	20.5	0.5

			Temperature			Temperature Association with Mortality		
**Country**	**City**	**Total Number of Deaths**	**5th Percentile Temperature**	**Median Temperature**	**95th Percentile Temperature**	**Minimum Mortality Temperature ^{0}C**	**Excess Death Fraction All Cold (%)**	**Excess Death Fraction All Heat (%)**
	Osorno	13008	4.4	10.5	17.7	20.1	19.2	-0.1
	Puerto Montt	13059	5.0	10.1	16.0	18.1	22.7	0.1
	Punta Arenas	9676	0.6	4.9	9.2	10.8	-9	0.1
	Quillota	12020	8.9	15.0	20.5	18.4	2.3	2.3
	Rancagua	20033	3.3	12.2	20.7	18.9	8.8	-0.5
	San Antonio	9559	8.5	13.7	18.5	19.6	13.9	-0.1
	Santiago de Chile	387651	5.8	14.5	22.1	23.5	15.2	0
	Talca	16898	6.7	14.2	23.1	20.9	10.1	-1.1
	Temuco	21901	4.9	11.1	18.8	17.3	13.4	0.8
	Valdivia	11453	5.1	10.7	17.7	20.2	18.9	-0.1
	Valparaíso-Viña del Mar	74162	9.1	14.0	18.7	19.6	14.5	0
**Costa Rica**	San José	64117	17.8	19.4	20.7	19.8	2.5	1.2
**El Salvador**	San Miguel	6601	24.6	27.1	30.1	26.6	0.6	3.2
	San Salvador	46465	21.9	23.8	25.8	26.5	36	-0.3
	Santa Ana	8751	20.7	23.2	25.6	23.8	0.8	-1.4
**Guatemala**	Ciudad de Guatemala	127016	16.3	18.8	20.8	20.1	1.7	-0.1
	Escuintla	7720	22.1	23.7	25.7	24.3	7.6	1.2
	Quetzaltenango	10688	12.8	14.5	15.7	16.5	3.5	0
**México**	Acapulco (Acapulco de Juárez) (ZM de Acapulco)	51823	24.0	25.8	28.0	25.1	0.8	3.7
	Acayucan (ZM de Acayucan)	6687	19.8	25.2	29.6	25.5	2.9	5.7
	Aguascalientes (ZM de Aguascalientes)	43701	12.3	18.4	23.6	20.4	9.5	1.4
	Campeche (San Francisco de Campeche)	14234	22.1	27.1	30.7	27.3	6.3	-0.6
	Cancún (ZM de Cancún)	21975	22.3	26.3	28.5	26.4	4.1	-1.7
	Celaya (ZM de Celaya)	41384	13.4	18.6	23.4	20.6	10.1	0.9

			Temperature			Temperature Association with Mortality		
Country	City	Total Number of Deaths	5th Percentile Temperature	Median Temperature	95th Percentile Temperature	Minimum Mortality Temperature ^{0}C	Excess Death Fraction All Cold (%)	Excess Death Fraction All Heat (%)
	Chetumal (Othón P. Blanco)	10943	22.2	26.1	28.7	25.6	-2.2	3.6
	Chihuahua (ZM de Chihuahua)	57734	7.4	19.6	28.4	22.9	6.4	1.2
	Chilpancingo (Chilpancingo de los Bravo)	9874	17.5	19.6	22.2	23	15.2	-0.1
	Ciudad Acuña	6412	8.5	22.9	32.3	26.2	16.2	-1.1
	Ciudad Juárez (Juárez) (ZM de Juárez)	82978	4.5	19.2	30.9	25	6.3	1.9
	Ciudad Obregón (Cajeme)	24693	15.8	25.3	33.0	28.3	6.7	1.7
	Ciudad Valles	9540	15.9	24.1	28.5	24.8	-0.5	1.9
	Ciudad Victoria	16362	12.9	22.5	27.8	23.9	10.4	-0.1
	Ciudad de México [Mexico City] (ZM del Valle de México)	1136469	11.0	15.2	18.8	17.4	5.7	0
	Ciudad del Carmen	10524	22.4	26.6	30.1	25.9	-0.4	0.1
	Coatzacoalcos (ZM de Coatzacoalcos)	19138	21.1	25.6	29.3	24.7	4.6	-0.3
	Colima (ZM Colima-Villa de Álvarez)	19304	20.4	23.1	25.8	23.9	3.6	1.8
	Cuauhtémoc	10374	5.1	15.2	22.9	18.8	2.1	1
	Cuautla (ZM de Cuautla)	24402	17.6	20.7	25.2	22.6	7	2.5
	Cuernavaca (ZM de Cuernavaca)	53405	16.3	19.7	23.6	21.1	4.2	1.6
	Culiacán (Culiacán Rosales)	42445	18.4	25.2	29.9	25.6	3.5	3.5
	Córdoba (ZM de Córdoba)	21392	14.2	19.8	22.6	20.4	3.8	1.6
	Delicias	9428	9.3	22.0	30.8	24.9	6.7	3.3
	Durango (Victoria de Durango)	31587	9.7	17.4	23.2	20.7	6.8	-0.2
	Ensenada	24874	9.8	16.6	24.9	22.4	4.2	0.1
	Fresnillo	11899	9.8	16.8	22.0	18.9	7.9	1.4
	Guadalajara* (ZM de Guadalajara)	237261	15.7	20.1	24.7	22.8	6.5	0.4
	Guanajuato	8520	12.1	17.5	22.3	20	13.6	0.8
	Guaymas (Heroica Guaymas) (ZM de Guaymas)	13274	16.0	24.4	32.3	29.7	4	2.1

			Temperature			Temperature Association with Mortality		
Country	City	Total Number of Deaths	5th Percentile Temperature	Median Temperature	95th Percentile Temperature	Minimum Mortality Temperature ^{0}C	Excess Death Fraction All Cold (%)	Excess Death Fraction All Heat (%)
	Hermosillo	40804	13.8	24.8	33.8	30.9	14.9	1.4
	Hidalgo del Parral	8168	8.4	18.6	26.0	21.5	13.1	-0.8
	Iguala (Iguala de la Independencia)	9537	22.5	25.4	30.3	24.2	0.6	0.1
	Irapuato	26970	14.3	19.1	24.4	21.4	6	1
	La Paz	13197	16.9	23.8	29.8	26.5	1.8	3.2
	La Piedad (La Piedad de Cabadas) (ZM de La Piedad-Pénjamo)	15474	14.4	18.8	24.1	21.2	-2.7	0.6
	León (León de los Aldama) (ZM de León)	73368	13.2	18.6	23.9	20.9	8.5	0
	Los Mochis (Ahome)	21853	17.4	25.2	31.5	27.7	10.5	0.7
	Manzanillo	7900	22.4	25.1	27.2	25.5	-4.4	2.9
	Matamoros (Heroica Matamoros) (ZM de Matamoros)	23848	13.8	24.8	29.6	25.4	2.6	0.9
	Mazatlán	24221	19.5	24.7	28.4	25.3	6.4	4
	Mexicali (ZM de Mexicali)	52603	12.4	24.3	36.6	33.5	7.7	2
	Minatitlán (ZM de Minatitlán)	21364	20.8	25.5	29.5	23.9	2.7	4.4
	Monclova (ZM Monclova-Frontera)	18667	9.2	22.6	30.4	24.9	15.5	3.2
	Monterrey (ZM de Monterrey)	213821	10.5	21.9	28.6	23.7	7.3	1.9
	Morelia (ZM de Morelia)	42626	13.0	16.7	20.6	18.4	2.6	0.7
	Mérida (ZM de Mérida)	57995	21.4	26.9	30.4	25.6	0.5	2.5
	Navojoa	10216	16.4	25.5	32.3	27.8	6.5	-0.3
	Nogales (Heroica Nogales)	9549	5.5	18.2	28.3	23.1	-5.7	0.3
	Nuevo Laredo (ZM de Nuevo Laredo)	20722	10.8	25.2	32.7	26.6	7.4	4.5
	Oaxaca (Oaxaca de Juárez) (ZM de Oaxaca)	30204	13.9	17.9	21.5	19.7	5.3	0.2
	Ocotlán (ZM de Ocotlán)	8589	16.0	20.2	24.8	22.7	7.6	3
	Orizaba (ZM de Orizaba)	29656	11.8	17.6	20.1	19.5	11	-0.1
	Pachuca (Pachuca de Soto) (ZM de Pachuca)	25139	8.8	13.6	17.1	18.4	3.9	0

			Temperature			Temperature Association with Mortality		
**Country**	**City**	**Total Number of Deaths**	**5th Percentile Temperature**	**Median Temperature**	**95th Percentile Temperature**	**Minimum Mortality Temperature ^{0}C**	**Excess Death Fraction All Cold (%)**	**Excess Death Fraction All Heat (%)**
	Piedras Negras (ZM Piedras Negras)	10140	9.3	23.9	33.1	26.9	6.8	0.5
	Playa del Carmen	4270	22.0	26.2	28.5	25.8	0.5	2.5
	Poza Rica de Hidalgo (ZM de Poza Rica)	36768	16.7	24.1	27.6	24.3	1.9	2.4
	Puebla (Heróica Puebla de Zaragoza) (ZM Puebla)	154580	12.5	16.1	19.3	20.5	10.1	0
	Puerto Vallarta (ZM de Puerto Vallarta)	15401	20.1	24.1	26.7	23.9	1.1	7.8
	Querétaro (Santiago de Querétaro) (ZM de Querétaro)	49050	12.3	17.8	22.4	19.7	5.5	-0.4
	Reynosa (ZM de Reynosa-Río Bravo)	34077	12.7	25.2	30.8	25.9	5.7	-0.5
	Rioverde (Río Verde) (ZM de Río Verde-Ciudad Fernández)	9291	12.1	20.5	25.0	21.7	11.2	2.3
	Salamanca	14250	14.5	19.4	24.6	21.5	6.6	0.7
	Saltillo (ZM de Saltillo)	39429	8.4	17.7	22.7	19.5	13.4	-0.3
	San Cristóbal de las Casas	7780	12.8	15.7	17.9	18.8	18.5	0
	San Francisco del Rincón (ZM de San Francisco del Rincón)	8593	13.6	18.8	24.1	21	5.1	-0.1
	San Juan Bautista Tuxtepec	7914	19.8	25.4	29.9	26.3	2.5	0.1
	San Juan del Río	10608	11.4	16.8	21.3	18.5	13.2	0.3
	San Luis Potosí (ZM de San Luis Potosí-Soledad de Graciano Sánchez)	50439	9.9	17.1	21.7	18.8	10.1	-0.6
	San Luis Río Colorado	9784	11.9	24.0	35.6	32.3	5.3	3.8
	Santo Domingo Tehuantepec (ZM de Tehuantepec)	9298	22.7	26.5	29.3	25.8	-4.5	7.4
	Tampico (ZM de Tampico)	49930	17.7	25.2	28.5	25.1	4.4	2.1
	Tapachula (Tapachula de Córdova y Ordóñez)	19418	22.9	24.5	26.4	24.9	-0.6	-1.4
	Tecomán (ZM Tecomán)	8960	23.1	25.6	27.8	25.8	0.1	3.3
	Tehuacán (ZM de Tehuacán)	14686	12.2	17.7	21.3	19.4	9.5	-0.9
	Tepic (ZM de Tepic)	21261	16.3	21.0	23.5	21.6	4.8	1.9
	Teziutlán (ZM de Teziutlán)	6796	9.2	14.6	17.5	18.8	31.2	-0.2
	Tianguistenco (ZM de Tianguistenco)	9026	8.9	12.6	15.2	16.5	-1.7	-0.1

			Temperature			Temperature Association with Mortality		
**Country**	**City**	**Total Number of Deaths**	**5th Percentile Temperature**	**Median Temperature**	**95th Percentile Temperature**	**Minimum Mortality Temperature ^{0}C**	**Excess Death Fraction All Cold (%)**	**Excess Death Fraction All Heat (%)**
	Tijuana* (ZM e Tijuana)	84415	9.9	16.1	23.5	21.9	9.3	0.2
	Tlaxcala (Tlaxcala de Xicohténcatl) (ZM de Tlaxcala-Apizaco)	24472	10.2	14.4	17.7	19	15.9	0
	Toluca (Toluca de Lerdo) (ZM de Toluca)	94146	9.5	13.2	16.2	17.5	-2.3	0
	Torreón (ZM de la Laguna)	69230	12.2	23.5	29.9	25	10.1	3.1
	Tula de Allende (ZM de Tula)	13777	10.6	15.7	19.8	21.2	21.5	0
	Tulancingo (Tulancingo de Bravo) (ZM de Tulancingo)	12001	8.3	13.8	16.8	18.1	-19.1	0
	Tuxtla Gutiérrez (ZM Tuxtla Gutiérrez)	32312	18.1	22.5	26.2	23.4	1.1	0.2
	Uriangato (ZM de Moroleón-Uriangato)	6582	14.0	18.1	23.0	21.2	3.1	-1.4
	Uruapan (Uruapan del Progreso)	17063	14.7	17.9	20.9	19.2	5.3	1.9
	Veracruz (ZM de Veracruz)	53342	20.3	25.6	29.0	25.1	-1	1.8
	Villahermosa (ZM de Villahermosa)	37912	21.4	26.2	31.1	25.1	-0.8	5.7
	Xalapa-Enríquez (Jalapa) (ZM de Xalapa)	38085	13.3	19.4	22.2	23.7	24.1	-0.1
	Zacatecas (ZM de Zacatecas-Guadalupe)	13948	9.7	16.2	20.9	18.2	5.6	0.8
	Zamora de Hidalgo (ZM de Zamora-Jacona)	14641	14.9	18.9	23.9	20.9	-4.7	2.2
**Panamá**	Colon	4737	24.8	25.7	26.7	27.3	-11	0.2
	David	4267	23.5	24.9	27.4	25.5	9.9	1.1
	Panama City	32524	24.7	25.8	27.1	25.4	-0.4	-0.7
**Perú**	Arequipa	29148	11.4	13.6	16.1	17.2	7.1	0
	Ayacucho	4833	9.4	11.6	13.4	14	26.5	-0.1
	Cajamarca	4120	10.2	11.6	13.0	13.6	64.1	-1.3
	Chiclayo	22919	18.0	21.1	25.7	23.8	3.1	0.2
	Chimbote	8851	16.2	19.2	24.0	22.5	20.4	-0.8
	Chincha Alta	6544	17.0	19.5	22.9	21.3	2.1	3.1
	Cusco (Cuzco)	7376	6.1	8.0	9.7	10.6	-7.3	0.1

			Temperature			Temperature Association with Mortality		
**Country**	**City**	**Total Number of Deaths**	**5th Percentile Temperature**	**Median Temperature**	**95th Percentile Temperature**	**Minimum Mortality Temperature ^{0}C**	**Excess Death Fraction All Cold (%)**	**Excess Death Fraction All Heat (%)**
	Huancayo	16284	6.9	8.7	10.2	10.8	-1.9	-0.1
	Huaraz	2971	6.4	8.0	9.5	10.1	77.6	-1
	Huánuco	6274	12.5	14.1	15.4	16	-0.9	-0.3
	Ica	10458	17.9	21.2	24.3	22.6	1.2	-0.1
	Iquitos	4663	23.9	25.5	27.4	24.8	1.7	16.8
	Juliaca	9400	5.6	8.4	11.1	12.2	1.4	0
	Lima	261923	15.9	18.6	22.2	21.1	6.9	0.5
	Pisco (incl. San Clemente)	3862	16.4	19.5	23.9	22.4	15.6	-0.9
	Piura	15607	20.6	23.9	27.6	25.7	6.8	1.1
	Pucallpa	9719	23.7	25.7	27.9	24.8	0.2	11.3
	Puno	4875	5.2	8.0	10.5	11.6	30.7	-0.4
	Sullana	8959	20.8	24.0	27.3	25.5	3.3	0.7
	Tacna	7822	13.8	17.4	20.8	19.3	-9.7	0.8
	Tarapoto	3535	22.5	24.1	26.0	24.3	-6.2	0.1
	Trujillo	30797	16.9	19.4	22.7	20.8	-1.8	1.6
	Tumbes	2518	22.7	24.8	26.5	25.3	-2.8	-0.7
