# Supplementary material for: City-level impact of extreme temperatures and mortality in Latin America
Source: Nat Med. Author manuscript; Available in PMC 2022 Sep 8. (PMC9388372; doi:10.1038/s41591-022-01872-6)
Supplement: Supplementary Infomration [file EMS151808-supplement-Supplementary_Information.pdf]

nature portfolio

Corresponding author(s): Josiah L. Kephart, PhD

Last updated by author(s): May 3, 2022

Reporting Summary

Nature Portfolio wishes to improve the reproducibility of the work that we publish. This form provides structure for consistency and transparency in reporting. For further information on Nature Portfolio policies, see our Editorial Policies and the Editorial Policy Checklist.

Statistics

For all statistical analyses, confirm that the following items are present in the figure legend, table legend, main text, or Methods section.

n/a	Confirmed	
☐	☒	The exact sample size (*n*) for each experimental group/condition, given as a discrete number and unit of measurement
☐	☒	A statement on whether measurements were taken from distinct samples or whether the same sample was measured repeatedly
☒	☐	The statistical test(s) used AND whether they are one- or two-sided<br>*Only common tests should be described solely by name; describe more complex techniques in the Methods section.*
☐	☒	A description of all covariates tested
☐	☒	A description of any assumptions or corrections, such as tests of normality and adjustment for multiple comparisons
☐	☒	A full description of the statistical parameters including central tendency (e.g. means) or other basic estimates (e.g. regression coefficient) AND variation (e.g. standard deviation) or associated estimates of uncertainty (e.g. confidence intervals)
☒	☐	For null hypothesis testing, the test statistic (e.g. *F*, *t*, *r*) with confidence intervals, effect sizes, degrees of freedom and *P* value noted<br>*Give P values as exact values whenever suitable.*
☒	☐	For Bayesian analysis, information on the choice of priors and Markov chain Monte Carlo settings
☒	☐	For hierarchical and complex designs, identification of the appropriate level for tests and full reporting of outcomes
☒	☐	Estimates of effect sizes (e.g. Cohen's *d*, Pearson's *r*), indicating how they were calculated

Our web collection on statistics for biologists contains articles on many of the points above.

Software and code

Policy information about availability of computer code

Data collection	All data collection was performed in R (v. 3.6.0) and Python (v. 3.9). The code repository for the estimation of city-specific daily temperature is available at https://github.com/Drexel-UHC/salurbal_heat.
Data analysis	All analyses were performed in R (v. 3.6.0) and modeling was done with the mvmeta (v. 1.0.3), dlnm (v. 2.3.9), and gnm (v. 1.1.1) packages. We used World Health Organization Global Health Estimate 2015 classifications to categorize cause of death by grouping International Classification of Diseases (v. 10) codes. Clustering of cities was performed using the factoextra (v. 1.0.7) package. The code repository for the main statistical analysis is available here: https://github.com/Drexel-UHC/SALURBAL-Heat-Study-MS85.

For manuscripts utilizing custom algorithms or software that are central to the research but not yet described in published literature, software must be made available to editors and reviewers. We strongly encourage code deposition in a community repository (e.g. GitHub). See the Nature Portfolio guidelines for submitting code & software for further information.

Data

Policy information about availability of data

All manuscripts must include a data availability statement. This statement should provide the following information, where applicable:

- Accession codes, unique identifiers, or web links for publicly available datasets
- A description of any restrictions on data availability
- For clinical datasets or third party data, please ensure that the statement adheres to our policy

City-specific temperature and mortality summaries and analysis outputs are freely available from an interactive app at https://drexel-uhc.shinyapps.io/MS85/. Links to the ERA5-Land, WorldPop, and Global Urban Footprint source datasets used to estimate population-weighted ambient temperature as well as final daily temperature outputs are available at https://github.com/Drexel-UHC/salurbal_heat. Vital registration and population data for Brazil, Chile, and Mexico were

downloaded from publicly available repositories from statistical agencies in each country. Vital registration and population data for Argentina, Costa Rica, El Salvador, Guatemala, Panama and Peru were obtained directly from statistical agencies in each country. A link to these agency websites can be accessed via https://drexel.edu/lac/data-evidence/data-acknowledgements/.

Field-specific reporting

Please select the one below that is the best fit for your research. If you are not sure, read the appropriate sections before making your selection.

☐ Life sciences ☒ Behavioural & social sciences ☐ Ecological, evolutionary & environmental sciences

For a reference copy of the document with all sections, see nature.com/documents/nr-reporting-summary-flat.pdf

Behavioural & social sciences study design

All studies must disclose on these points even when the disclosure is negative.

Study description	This quantitative analysis is a time-series/longitudinal ecological analysis of city-level associations between daily ambient temperature and daily mortality, with stratifications by age and cause of death groupings.
Research sample	Our sample consists of all cities with available daily mortality data within the SALURBAL project (N=326 cities in this study from a total of 371 cities in the SALURBAL project). The SALURBAL project has compiled and harmonized data on environmental, social, and health characteristics for all cities of 100,000 residents or more in 11 Latin American countries. Cities in SALURBAL were defined as urban agglomerations composed of clusters of administrative units encompassing the visually apparent urban built-up area as identified using satellite imagery. In this analysis, we include 326 cities in Argentina, Brazil, Chile, Costa Rica, El Salvador, Guatemala, Mexico, Panama, and Peru. Cities in Colombia and Nicaragua were excluded due to the limited availability of daily mortality data. Daily mortality counts were aggregated for each city between 2002-2015, representing a total of 15,431,532 deaths among ≈ 2.9 billion person-years of risk.
Sampling strategy	We included all cities of at least 100,000 residents, as of 2010, within nine Latin American countries. This threshold was selected to capture cities from a range of population sizes, from small cities to megacities. We included all recorded deaths, without exclusion for age, sex, or otherwise.
Data collection	We used ERA5-Land climate reanalysis and established population density maps to estimate population-weighted daily ambient temperature for each city during the study period. Mortality counts were compiled directly from governmental vital records agencies and aggregated to city-level counts. Data collection was performed by a researcher not directly involved with the main statistical analysis. Researchers involved in data collection were not blinded to the study hypothesis.
Timing	The observation period varied by country depending on availability of mortality data, with a country-specific observation period of 4-14 years duration, between the range 2002-2015.
Data exclusions	No data was excluded in the analysis.
Non-participation	All deaths recorded in the vital registration systems were included.
Randomization	There was no randomization in this observational study. The models conditioned on strata defined by day of the week, month and year, offering strong control for seasonality and secular changes, and yielding inferences based on within-city short-term temperature variability.

Reporting for specific materials, systems and methods

We require information from authors about some types of materials, experimental systems and methods used in many studies. Here, indicate whether each material, system or method listed is relevant to your study. If you are not sure if a list item applies to your research, read the appropriate section before selecting a response.

Materials & experimental systems

n/a	Involved in the study
☒	☐ Antibodies
☒	☐ Eukaryotic cell lines
☒	☐ Palaeontology and archaeology
☒	☐ Animals and other organisms
☒	☐ Human research participants
☒	☐ Clinical data
☒	☐ Dual use research of concern

Methods

n/a	Involved in the study
☒	☐ ChIP-seq
☒	☐ Flow cytometry
☒	☐ MRI-based neuroimaging
